# Genome structure variation analyses of peach reveal population dynamics and a 1.67 Mb causal inversion for fruit shape

**DOI:** 10.1186/s13059-020-02239-1

**Published:** 2021-01-05

**Authors:** Jiantao Guan, Yaoguang Xu, Yang Yu, Jun Fu, Fei Ren, Jiying Guo, Jianbo Zhao, Quan Jiang, Jianhua Wei, Hua Xie

**Affiliations:** 1 Beijing Agro-Biotechnology Research Center, Beijing Academy of Agriculture and Forestry Sciences, Beijing, People’s Republic of China; 2 Beijing Key Laboratory of Agricultural Genetic Resources and Biotechnology, Beijing, People’s Republic of China; 3 Institute of Forestry and Pomology, Beijing Academy of Agriculture and Forestry Sciences, Beijing, People’s Republic of China

**Keywords:** Peach genome assembly, Structure variation, Population dynamics, Genome-wide association study, Large heterozygous inversion, *PpOFP2*, Fruit shape

## Abstract

**Background:**

Structural variations (SVs), a major resource of genomic variation, can have profound consequences on phenotypic variation, yet the impacts of SVs remain largely unexplored in crops.

**Results:**

Here, we generate a high-quality de novo genome assembly for a flat-fruit peach cultivar and produce a comprehensive SV map for peach, as a high proportion of genomic sequence is occupied by heterozygous SVs in the peach genome. We conduct population-level analyses that indicate SVs have undergone strong purifying selection during peach domestication, and find evidence of positive selection, with a significant preference for upstream and intronic regions during later peach improvement. We perform a SV-based GWAS that identifies a large 1.67-Mb heterozygous inversion that segregates perfectly with flat-fruit shape. Mechanistically, this derived allele alters the expression of the *PpOFP2* gene positioned near the proximal breakpoint of the inversion, and we confirm in transgenic tomatoes that *PpOFP2* is causal for flat-fruit shape.

**Conclusions:**

Thus, beyond introducing new genomics resources for peach research, our study illustrates how focusing on SV data can drive basic functional discoveries in plant science.

## Background

Structural variations (SVs) including deletions, insertions, duplications, and inversions, a major resource of genomic variation, are known to strongly impact phenotypes [1–4]. In human genomics research, major breakthroughs have shown that SVs are associated with phenotypes more frequently than smaller genomic variants (e.g., SNPs and InDels) [5]. There are also demonstrated cases in which SVs substantially contribute to human cancers and other various diseases and disorders [6–8] by affecting relevant gene dosage, function(s), and/or regulation [1, 3, 9, 10]. In plants, molecular genetic analyses have highlighted the functional importance of SVs on protein-coding and flanking noncoding regions of loci/genes linked to agriculturally important traits, e.g. grain size [11, 12], fruit shape [13], fruit weight [2], and fruit color [14, 15]. However, despite these significant advances, the contribution of SVs, especially large chromosomal rearrangements, to specific traits remains largely uncharacterized in plant organisms. This is particularly true for clonally propagated crops such as grapevine [16, 17] and cassava [18], which are known to accumulate heterozygous somatic mutations. Understanding the genomic context of SVs (and especially heterozygous SVs) can help elucidate the basis of phenotypic diversity.

Peach (*Prunus persica*) is the third-ranking clonally propagated temperate tree crop (after apple and pear) in terms of annual global production (FAOSTAT). Peach has been widely studied as a model for the genus *Prunus* and for other Rosaceae perennial fruit trees [19, 20]. However, the characterization of genome-wide SVs and their potential phenotypic impacts remains a largely unexplored area. It is notable that the heterozygous SVs contribute to functional impacts during domestication [17], while the currently available peach reference genome generated from a double haploid genotype of peach cv. Lovell [21, 22] has resulted in the loss of heterozygous genomic information. Therefore, a high-quality heterozygous peach genome is currently needed for genome-wide mining of SVs that may impact desirable traits.

Peach originated in China over 2,000,000 years ago [23, 24] and wide phenotypic variations in fruit size, shape, color, texture, and flavor have been retained throughout its 8500-year domestication [25]. Notably, SVs were found to contribute to distinct peach phenotypic differences in Mendelian fruit traits such as flesh color [26], skin pubescence [27], flesh softening and adhesion [28], and stone texture [29]. Therefore, initiating a study of high-confidence SVs in peach populations can advance our understanding of a wide range of agronomically desirable traits.

Fruit shape is a highly valued agronomic trait in cultivated peach, and inheritance of this qualitative fruit shape was initially described to be under the control of a single Mendelian factor [30]. This dominant “*S*” for “saucer-shaped” locus was later mapped to the distal part of chromosome 6 by linkage analysis [31–34]. Flat fruits were found to carry the heterozygous dominant “*Ss*” genotype, whereas round fruits retain the ancestral homozygous recessive “*ss*” genotype; note that the homozygous dominant mutational genotype “*SS*” produces aborted fruits during early fruit development [31, 33, 35]. Using SNP-based GWAS (genome-wide association study), multiple strongly fruit-shape-associated SNP signals have been mapped to the “*S*” locus [36–38]. Candidate flat-fruit shape genes at this locus include *PpCAD1* (constitutively activated cell death 1) [37] and *LRR-RLK* (leucine-rich receptor-like kinase) [35]. However, a recent population-scale study suggested that the reported mutations for these two genes are apparently insufficient to explain flat fruit shape traits in some cultivars [39]. Thus, genetic basis underlying this trait merits further investigation.

Here, we generated a high-quality de novo genome assembly for a flat peach cultivar using high-depth PacBio long-read data complemented with Illumina short-read data and used it as the basis to reveal a high proportion of genomic sequence occupied by heterozygous SVs in peach genome. We produced a comprehensive SV map for peach across 149 peach accessions, characterized SV hotspots at population-scale, and provided insights into the selection of SVs during peach domestication and improvement. SV-based GWAS facilitate our identification of a 1.67-Mb heterozygous inversion that resulted in the upregulation of its adjacent gene *PpOFP2* to cause flat fruit shape. Thus, our study presents a high-quality peach genome assembly and a comprehensive SV map that substantially deepens our population-level understanding about the long-term functional impacts of SVs in peach and also illustrates an example for how SV data can be profitably used in plant science to gain basic functional insights.

## Results

### Genome assembly and annotation of RYP1

**Fig. 1:**
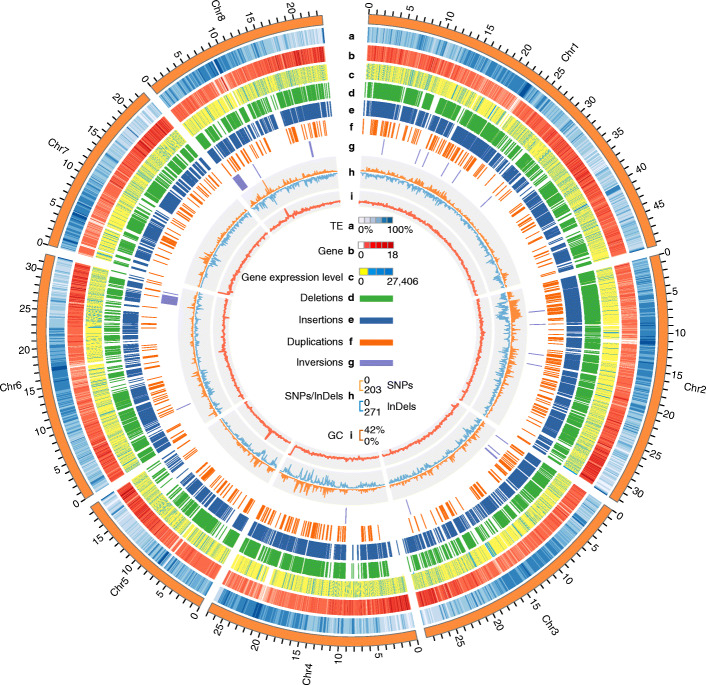
Heterozygous genomic variants and other features across the RYP1 genome. Tracks (outer to inner circles) indicate the following: **a**, **b** Transposable-element (TE) (**a**) and gene (**b**) density (window size of 100 kb). **c** Gene expression level (FPKM; the highest expression of different sequenced tissues - see Table S4 for details). **d**–**h** Heterozygous deletions (**d**), insertions (**e**), duplications (**f**), and inversions (**g**) identified by high-depth (589.74×) PacBio long-read data, and SNPs/InDels (**h**) identified by 147.97× coverage Illumina short-read data (the outer and inner layers respectively indicate SNPs and InDels density in a 100 kb window size). **i** GC content (window size of 100 kb). The outer orange circle represents the chromosome length (Mb) for the RYP1 genome assembly

The genome of Rui You Pan 1 (RYP1) was de novo assembled using 140.84 Gb PacBio long reads (~ 589.74× coverage) and 35.37 Gb Illumina short reads (~ 147.97× coverage) using the pipeline detailed in Additional file 1: Fig. S1 and Additional file 2: Table S1. We assembled the PacBio long reads from RYP1 into contigs using the Canu pipeline (version 1.8) [40] and then improved contigs into 87 super contigs by employing the HERA algorithm [41]. The super contigs were corrected with the Illumina short reads from RYP1 and total assembled length reached 239.1 Mb, with the longest super contig being 25.9 Mb (Table 1). Given the generally large size of these contigs (contig N50 size of 11.5 Mb), a total of 62 contigs, which accounted for 98.3% (~ 235.0 Mb) of the total assembled sequences, were anchored into chromosome-level pseudomolecules based on homology to the current peach Lovell v2.0 reference genome [22] (Fig. 1, Additional file 2: Table S2). The number of unplaced contigs in our RYP1 assembly is 25, comprising a total of 4.05 Mb, whereas the number of unplaced scaffolds in the Lovell v2.0 assembly is 183, representing a total of 1.72 Mb (Additional file 2: Table S3). The coverage and contiguity of the RYP1 genome assembly were both improved compared with the Lovell v2.0 genome; consistently, there were as few as 54 gaps in the RYP1 genome assembly compared to the Lovell v2.0 genome (Table 1).

**Table 1:** Summary of statistics for the RYP1 genome assembly compared with the published Lovell v2.0 reference genome

Category	RYP1	Lovell v2.0
Total assembly size (Mb)	239.1	227.4^b^
Number of contigs	87	2525^b^
Largest contigs (Mb)	25.9	1.5^b^
Contig N50	11.5 Mb	255.4 kb^b^
Sequences anchored to chromosomes (Mb)	235.0	225.7^b^
Genomic GC content (%)	37.6	37.0
Number of gaps	54	1828
Complete BUSCOs (%)^a^	97.4	97.6
LTR assembly index, LAI	22.33	21.29
Intact LTR length (Mb)/number	10.50/1656	8.84/1416
Repetitive sequences (Mb)/(%)	115.01/48.11	101.99/44.85
Protein-coding genes	32,604	31,972^b^
Transcripts	37,827	47,089
Average gene length (bp)	2223	2215

^a^The analysis from comparisons with the embryophyta_odb9 database

^b^The statistic values taken from the previous publication [22]

Next, the quality of the assembled RYP1 genome was assessed using the following four strategies. First, we examined the consistency of physical and genetic maps: all 3092 markers from a recently published multi-population consensus genetic linkage map [42] were used in this analysis, and among these, 2257 markers could be uniquely aligned to our RYP1 genome; moreover, a high proportion (93.09%) of the uniquely mapped markers were found to be located at their expected position (Additional file 1: Fig. S2). Second, the accuracy and completeness of the RYP1 genome was supported by a high mapping rate (98.82%) for the Illumina whole-genome short reads (Additional file 2: Table S1). Third, we used the LTR Assembly Index (LAI) [43] to evaluate the continuity of our RYP1 genome assembly, which reached a “gold standard level” and had a high LAI score (22.33) which exceeded that of the Lovell v2.0 genome (21.29) (Table 1, Additional file 1: Fig. S3). Fourth, approximately 97.4% (1402 of 1440) of complete BUSCO hits were detected in our assembly, similar to the level obtained for the Lovell v2.0 genome (97.6%; 1405 of 1440) (Table 1, Additional file 2: Table S4). Collectively, these multiple lines of evidence attest to the high-quality of our de novo RYP1 genome assembly, supporting its utility as an excellent reference for genomic-variation mining and for comparative studies in peach.

We next conducted ab initio gene prediction using an integrative strategy combining in silico de novo gene prediction, protein-based homology searches, and transcript data from RNA sequencing analysis of various tissues (Additional file 2: Table S5). We were able to annotate a total of 32,604 protein-coding genes and 37,827 transcripts, with an average sequence length of 2223 bp, a length similar to that (2215 bp) for a re-annotated version of the Lovell v2.0 genome using the same pipeline (Table 1). Of the protein-coding genes, 93.9% (1352 of 1440) complete BUSCOs were found (Additional file 2: Table S4), and 89.50% were successfully functionally annotated with five public database resources (Additional file 2: Table S6).

We identified repeat sequences of RYP1 genome assembly using RepeatMasker (see Methods). A total of 115.01 Mb of repetitive elements occupying 48.11% of the RYP1 genome were thusly annotated (Table 1), including retrotransposons (20.47%), DNA transposons (13.23%), and other repeats (14.42%) (Additional file 2: Table S7). We also re-annotated the repeat sequences of the Lovell v2.0 genome [22] with the same approach: ~ 13.02 Mb more total repeat sequences were identified in our RYP1 genome assembly (115.01 Mb) compared to the Lovell v2.0 genome assembly (101.99 Mb) (Table 1, Additional file 2: Table S7). Examining repeat sequence differences in detail, the total length/number of intact LTRs was increased by ~ 1.66 Mb/240 in the RYP1 genome (10.50 Mb/1656) compared to the Lovell v2.0 genome (8.84 Mb/1416) (Table 1), highlighting that our RYP1 genome assembly provides additional, accurate genome information for chromosomal regions with high repeat sequence content.

### Genomic variation between the RYP1 and Lovell v2.0 genomes

Having obtained a high-quality genome sequence for RYP1, we first conducted a global genome comparison of RYP1 and Lovell v2.0 to assess genomic variations in peach. For phenotypic context, RYP1 is a flat, low acid, white flesh, nectarine cultivar that is popular in China, whereas Lovell has round, acid, yellow flesh color fruit and its glabrous skin (Additional file 3: Table S8), and has also been widely used as a rootstock [44]. Analysis with the MUMmer program [45] highlighted the excellent collinearity between the RYP1 and Lovell v2.0 genomes (Additional file 1: Fig. S4): approximately 88.67% (211.97 Mb) of the RYP1 genome sequences matched one-to-one syntenic blocks with 92.74% (210.90 Mb) of the Lovell v2.0 genome. The 11.33% (~ 27.08 Mb long) nonsyntenic sequences in the RYP1 genome (with the exclusion of gaps in the two genome assemblies) were mostly repeat sequences (64.39%). There were 971 and 988 specific presence/absence variations (PAVs) identified between the RYP1 and Lovell v2.0 genomes, respectively (Additional file 3: Table S9).

OrthoFinder pipeline [46] analysis revealed 31,347 RYP1 and 30,815 Lovell genes as corresponding orthologous genes, whereas 103 and 87 specific orthogroups containing 772 and 580 genes were identified as specific to the RYP1 and the Lovell genomes, respectively (Additional file 2: Table S10). In addition, we also found that 992 and 1590 expansion orthogroups containing 3571 and 5096 genes displayed higher copy numbers of genes in the Lovell v2.0 or the RYP1 genomes, respectively, (Additional file 2: Table S10). KEGG (Kyoto Encyclopedia of Genes and Genomes) enrichment analysis was further performed for the specific and expansion orthogroups. The results showed RYP1-specific enrichment for the “fructose and mannose metabolism” pathway (ko00051) (Additional file 1: Fig. S5a, b). Detailed examination of the genes in this pathway showed that two genes related to fructose metabolism, sorbitol dehydrogenase (*SDH*) [47] and pyrophosphate fructose-6-phosphate 1-phosphotransferase subunit beta (*PFPβ*) [48], had higher copy numbers in the RYP1 genome (3 and 4 copies) than in the Lovell v2.0 genome (2 and 3) (Additional file 1: Fig. S5c).

Considering that gene duplication events are known to represent a major mechanism underlying phenotypic change via gene dosage effects or gene functional divergence [49], we identified the singleton genes: there were 4713 in the RYP1 genome and 4944 in the Lovell genome (Additional file 2: Table S11). We also noted that RYP1 had relatively more segmental duplications (5271), but fewer dispersed duplicated genes (15,750) compared with Lovell v2.0. We identified 3853 tandem duplicated genes in 1652 tandem gene copy clusters of the RYP1 genome.

### Hidden heterozygous SVs of the peach genome

Recent progress in genomics research is making it increasingly clear that SVs exert large functional impacts [2, 50], and to date, SVs in peach remain little explored. Given the high continuity of our RYP1 genome assembly and its basis in high-depth PacBio long-read data, we were able to identify high-confidence SVs (> 30 bp) using an integrated method combining three independent analysis pipelines (see Methods). We discovered a total of 11,480 heterozygous SVs, including 5182 insertions, 5578 deletions, 699 duplications, and 21 inversions, accounting for a total sequence length of ~ 23.15 Mb (Fig. 1, Additional file 2: Table S12).

We assessed the heterozygosity of the RYP1 genome using its heterozygous 504,069 SNPs (Additional file 2: Table S12). The heterozygosity was estimated to be 0.22%, which was consistent with the reported value in peach [24], but was much lower than in grape (7%) [51] or pear (~ 1.02%) [52]. Interestingly, compared with low SNP heterozygosity inferring a low proportion of heterozygous sequences for the RYP1 genome, the heterozygous SVs occupied up to 9.68% of the whole genome, which was comparable to the level of heterozygous SVs in grape (~ 15%) [17]. In addition, the RYP1 heterozygous SVs overlapped with 2244 protein-coding genes, accounting for 6.88% of all annotated genes. At minimum, this heterozygosity analysis provides a wealth of evolutionary evidence that many SVs have been retained in a heterozygous state in peach, implying a strong potential for SV-mediated biological consequences in this species. Thus, cataloging peach SVs can support genomic explorations into the functional impacts of heterozygous SVs in diverse peach accessions.

### Construction of a comprehensive SV map in peach

**Fig. 2:**
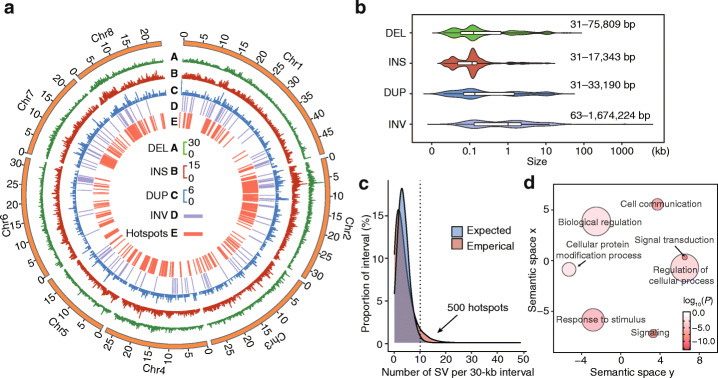
Peach SV map and hotspots. **a** The tracks from A to E of the circos plot respectively represent the density of deletions (DEL), insertions (INS), and duplications (DUP) in a 100-kb sliding window, and inversions (INV), and hotspots in the peach SV map. **b** Size distribution of different types of SVs. In the box plots, central line: median values; bounds of box: 25th and 75th percentiles; whiskers: 1.5 * IQR (IQR: the interquartile range between the 25th and 75th percentile). **c** The expected and empirical probability distributions of SVs in 30-kb long intervals were estimated assuming a Poisson distribution. A total of 500 intervals with more than 10 SVs in the empirical distribution were considered SV hotspots based if they fell within the 99th percentile or higher of the expected distributions. **d** Scatter plot of enriched GO terms under the ‘biological process’ category for the genes located within hotspot intervals. *p* < 0.05, calculated using Fisher’s exact test. Color intensity reflects the significance of enrichment, with darker colors representing lower *P* values. Circle sizes represent the frequency of each enriched GO term in the Gene Ontology Annotation database (bubbles of more general terms are larger)

Given the strong potential for biological consequences resulting from SVs, we constructed a comprehensive SV map for the peach genome by first identifying the SVs using resequencing short-read data (average 31.52×) for a total of 149 diverse *P. persica* accessions from major peach-producing areas, including north, northwest, south, southwest China, Japan, the Americas, and Europe [53] (full details for the accessions comprising the germplasm diversity panel are presented in Additional file 3: Table S13). Note that phylogenetic tree and population structure analyses both support the division of the panel into landraces, oriental modern cultivars, and occidental modern cultivars [36] (Additional file 1: Fig. S6). We built the peach SV map by combining the aforementioned SVs detected from RYP1 and the output from the Manta analysis of the short-read data from those 149 accessions (see the “Methods” section). The SV map comprised a total of 27,734 SVs (> 30 bp), including 15,138 deletions, 10,882 insertions, 1558 duplications, and 156 inversions, which together covered about 16.10% (~ 38.49 Mb) of the total RYP1 genome (Table 2, Fig. 2a, b, Additional file 2: Table S14). In addition, 83.11% of these SVs were evident in at least 90% of the 149 accessions (Additional file 1: Fig. S7). Through PCR-based validation (see the “Methods” section), we estimated that the false discovery rate of total SVs, deletions, and insertions was 5.77%, 5.69%, and 5.94% (Additional file 3: Table S15), which was comparable with other studies of maize and cucumber [54, 55]. A principal component analyses (PCA) based on the SV data yielded a highly coincident pattern to the PCA model obtained based on whole-genome SNP data for the same accession panel (Additional file 1: Fig. S8), findings underscoring that these are high-confidence SVs suitable for use in peach functional genomic studies.

**Table 2:** Summary and annotation statistics for different types of SVs in peach

Category	Deletions	Insertions	Duplications	Inversions	Total
Number	15,138	10,882	1558	156	27,734
Size (bp)	25,889,284	6,484,273	3,896,898	2,222,324	38,492,779
CDS	2539 (2820)	474 (434)	409 (628)	63 (417)	3485 (4299)
Full CDS	1233 (1562)	0 (0)	173 (322)	30 (378)	1436 (2262)
Partial CDS	1306 (1258)	474 (434)	236 (306)	33 (39)	2049 (2037)
Intron	1081 (933)	1156 (989)	123 (123)	9 (8)	2369 (2053)
Upstream (< 1 kb)	2209 (2177)	2030 (1955)	180 (186)	16 (16)	4435 (4334)
Downstream (< 1 kb)	1532 (1865)	1457 (1717)	147 (194)	8 (14)	3144 (3790)
Intergenic	7767	5754	697	60	14,218

Values inside parentheses indicate the number of genes overlapping with the number of SVs

Our first analysis based on the SV map was an assessment of the potential functional impacts of SVs based on the positions of SVs relative to gene models. Most SVs (51.27%, 14,218 of 27,734) are located in intergenic regions, while 3485 SVs affected coding sequences (CDS) of up to 4299 protein-coding genes (Table 2). The CDS regions for a total of 1562 genes were entirely deleted, 322 genes were duplicated, and 378 genes were positioned fully within inverted regions. We also noted that a significantly higher proportion of SVs were located in putative promoter regions (upstream (< 1 kb); 4435 SVs) as compared to SVs located within CDS regions (3485 SVs, *P* < 0.001, Fisher’s exact test) (Table 2), consistent with a previous study of 3000 rice genomes [4]. A GO-based analysis indicated enrichment for the “signal transduction” and “response to stimulus” terms for genes with SVs overlapping their CDS regions; for genes with SVs in their putative promoters, there was predicted enrichment for GO terms including “ADP binding,” “small molecule binding,” and “peptidase regulator activity” (*P* < 0.05, Fisher’s exact test) (Additional file 3: Table S16). Given that an SV occurring within a CDS is very likely to result in pronounced “loss-of-function” effect, we speculate that this type of SVs would likely have faced very strong purifying selection during evolution, and thus less abundant SVs were retained in CDS regions compared to that located in putative promoter regions.

We next identified the SV hotspots in the peach genome by comparing the expected and empirical distributions of SVs (see the “Methods” section; Fig. 2c). A total of 500 intervals (interval size of 30 kb) containing ≥ 10 SVs in the empirical distribution were characterized as SV hotspots: these intervals fell within the 99th percentile (or higher) of the expected distributions. Relative to non-hotspots regions, the SV hotspots were significantly enriched for segmental duplications and for non-allelic homologous recombination (NAHR) formation events (Additional file 1: Fig. S9). These findings based on our peach SV data mirror the previously reported conclusion that SV hotspots tend to coincide with segmental duplications and that duplication-mediated NAHR mechanisms have contributed strongly to the formation of SV hotspots [56, 57].

Seeking to make functional inferences based on our SV hotspot data for peach, we conducted functional enrichment analysis for genes positioned within the SV hotspots regions. The 1767 genes in our defined SV hotspot regions were enriched for predicted functional annotations related to terms including “signal transduction,” “response to stimulus,” “signaling,” “response to stimulus,” and “cell communications” (Fig. 2d). Notably, three consecutive hotspot intervals located at Chr2: 15.42–15.51 Mb contained a cluster with 9 ortholog genes of wheat *LRK10* gene which was cloned from the basis of *Lr10* disease-resistance locus and encodes receptor-like protein kinase [58, 59]; the genic regions (including upstream, downstream, intronic, and exonic regions) of these 9 genes in this hotspot overlapped with at least one SV (Additional file 3: Table S17). Thus, SV hotspots have apparently contributed to the ability of peach to overcome stress-related and environmental challenges, a situation similar to a report about Arabidopsis [60].

### Selection of SVs during domestication and improvement

**Fig. 3:**
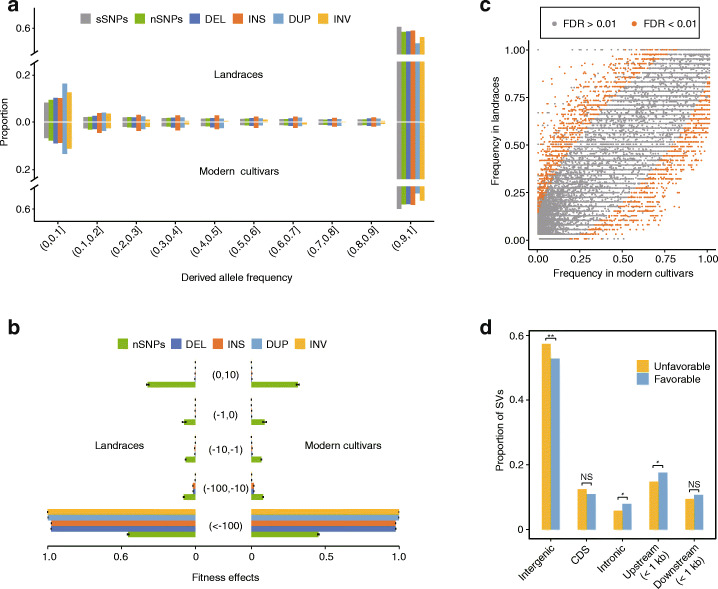
SVs are deleterious and underwent preferential selection during improvement. **a** The proportion of SVs with different unfolded site frequency spectrum (SFS) frequencies compared to presumably neutral sSNPs (synonymous SNPs), nSNPs (non-synonymous SNPs), and SVs (DEL, INS, DUP, and INV) for landraces (top) (*n* = 41) and modern cultivars (bottom) (*n* = 108) compared with *P. kansuensis* accessions (*n* = 37) as the outgroup. **b** The inferred distribution of fitness effects for SVs and nSNPs in landraces (left) and modern cultivars (right) based on 100 bootstrap replicates. Error bars indicate the mean ± 95% CI. **c** Scatter plots showing gene occurrence frequencies in landraces and modern cultivars. The significantly selected genes harbored adjusted *P* values (*FDR*) smaller than 0.01. **d** Proportion of SVs overlapping with different regions relative to gene models, including intergenic, CDS, intronic, upstream (< 1 kb), and downstream (< 1 kb) regions for unfavorable and favorable SVs during improvement, respectively. NS (not significant) *P* > 0.05, *P < 0.05, ** *P* < 0.01 in Fisher’s exact test

Seeking SV-related insights about peach population dynamics, we first analyzed the purifying selection against SVs during domestication by computing the unfolded site frequency spectrum (SFS) for each cultivated *P. persica* (landrace and modern cultivar), using *P. kansuensis* (*n* = 37)—the closest wild relative species of this cultivated species [24]—as the outgroup. Although previous studies have established that genetic drift tends to increase the extent of fixed-derived variants in derived populations (both in landraces and cultivars) [61], we found that all types of SVs exhibited leftward shifts in their SFSs relative to synonymous SNPs (sSNPs) (Fig. 3a); this trend is similar to a report for grapevine [17], with the largest shifts observed for insertions and inversions (*P* < 0.05, Wilcoxon test), suggesting that most of the peach SVs were deleterious.

To further estimate the strength of the purifying selection against SVs, we inferred the distribution of fitness effects in landraces and modern cultivars using sSNPs as a neutral control. The results indicated that peach SVs have undergone an extremely elevated extent of purifying selection compared to non-synonymous SNPs (nSNPs) (Fig. 3b). Note that this situation in peach differs markedly from the conclusions for grapevine, wherein SVs exhibited a comparable extent of purifying selection relative to nSNPs [17]. We speculate that this difference likely reflects peach’s high level of self-compatibility [62] which increases the chance of forming homozygous deleterious SVs, and thus typically accelerates the process of purifying selection. However, despite the strong purifying selection against SVs, we also observed accumulation of heterozygous SVs in modern cultivars during improvement, as supported by the significantly increased heterozygosity ratio we observed for SVs in modern cultivar vs. those for the landraces (*P* < 0.001, Wilcoxon test, Additional file 1: Fig. S10). We speculate that this accumulation of heterozygous SVs in modern cultivars can be attributed to crossbreeding and subsequent clonal propagation of these accessions, which would have retained these SVs in a heterozygous state during peach improvement efforts.

We further compared the total number of SVs for the landraces and modern cultivars (24,918 vs. 24,668) and found that their high proportions for shared SVs are consistent with the results from our SNP-based analysis (Additional file 1: Fig. S11). We also detected a slight reduction in the genetic diversity of modern cultivars estimated as the ratio of *θ*_π landraces_ /*θ*_π modern cultivars_ (0.2359/0.2035 = 1.16) from their SVs, again similar to their ratio based on their SNP data (0.2369/0.2000 = 1.18). These results support the previous conjecture that the genetic diversity of peach has not been substantially impacted throughout the course of peach improvement [24].

Given that modern peach cultivars exhibit agriculturally attractive phenotypes, we conceptualized all of the SVs as “improvement unfavorable” or “improvement favorable” based on their preferential frequency in landraces or modern cultivars, respectively; thus, a total of 1992 unfavorable and 1389 favorable SVs were identified (FDR < 0.01, Fisher’s exact test) (Fig. 3c, Additional file 3: Table S18). We also explored the distribution of these two types of SVs in intergenic, CDS, intronic, and upstream (< 1 kb) and downstream (< 1 kb) regions across the peach genome. An obvious trend was that the frequency of improvement unfavorable SVs was significantly enriched for intergenic regions whereas the frequency of improvement favorable SVs was significantly enriched in upstream regions (putative promoter regions) and in intronic regions (Fig. 3d).

### A 1.67-Mb heterozygous inversion is the causal variation for flat-fruit shape

**Fig. 4:**
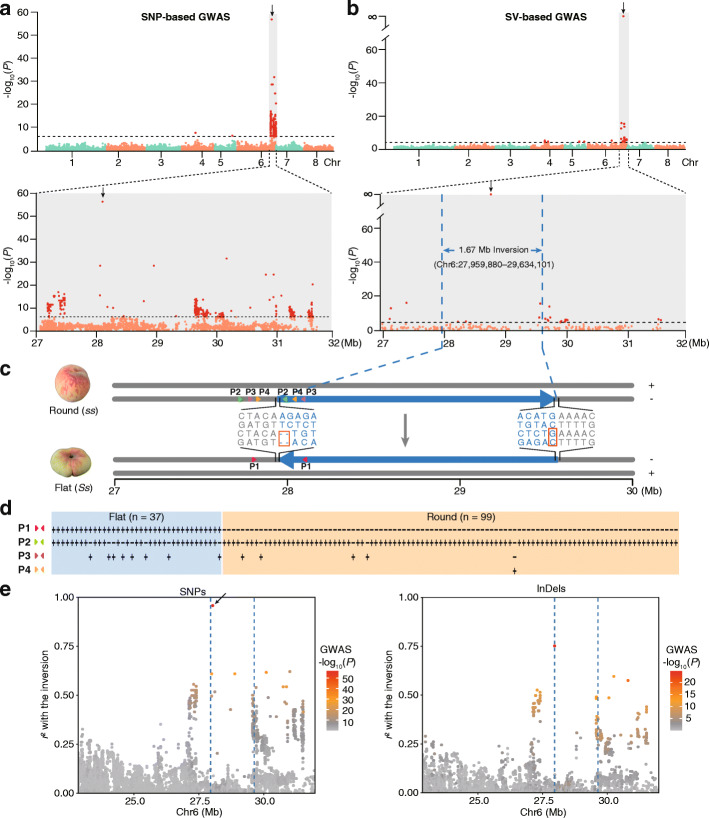
A 1.67-Mb heterozygous inversion is the causal variation for flat fruit shape. **a**, **b** Genome-wide (top) and regional (bottom) Manhattan plots using GWAS for fruit shape (round/flat) based on SNPs (**a**) and SVs (**b**). The regional plots showed the significant region (27.0–31.6 Mb) of Chr6. Each dot in plot (**a**) represents a SNP and in plot (**b**) represents the middle position of an SV. The black arrow in plot (**a**) points to the lead SNP (Chr6: 28,036,986) in this study, which was also reported in previous study [36]. The black arrow in plot (**b**) points to the middle position of the 1.67-Mb inversion (Chr6: 27,959,880–29,634,101). Horizontal dashed black lines correspond to the Bonferroni-corrected significance threshold for SNPs (5.94) and SVs (4.23) in (**a**) and (**b**), respectively. Vertical dashed blue lines correspond to the breakpoints of the inversion. **c** The heterozygous inversion in flat peach was formed by a NHEJ (non-homologous end-joining) mechanism accompanying two mis-repair events: the deletion of two base-pairs and the insertion of one base-pair respectively at the breakpoints highlighted in red dashed rectangles. **d** The association between the 1.67-Mb inversion and fruit shape was further verified in a diverse population that included 37 flat accessions (blue background) and 99 round accessions (orange background) using four pairs of primers (P1 for the sequences flanking the proximal breakpoint of the derived allele, and P2, P3, and P4 for those of the ancestral allele). The plus and minus signs represent the presence or absence of PCR products, respectively. **e** Linkage disequilibrium (LD) between the 1.67-Mb inversion and SNPs (left) or InDels (right) in the 23.00–31.98 Mb region of Chr6. Disequilibrium is measured as *r*^*2*^. The black arrow points to the lead SNP (Chr6: 28,036,986). Inversion breakpoints are depicted as vertical dashed blue lines. The color of SNPs and InDels with at least 5% minor allele frequency is shown as the *P* values of GWAS analysis, with red colors representing lower *P* values

Our genome-wide SVs map for peach represents a powerful data resource for finding causal genes that control agriculturally important traits and offers a unique opportunity to identify phenotypic effects caused by SVs. To explore the genetic basis underlying flat fruit shape—the most obvious RYP1 phenotypic trait, we performed GWAS analysis using our genome-wide data for the germplasm diversity panel, including 869,345 SNPs, 191,279 small InDels (≤ 30 bp), and 16,883 SVs (MAF ≥ 0.05) (Fig. 4a, b, Additional file 1: Fig. S12). This linear mixed model GWAS identified a total of 880, 167, and 33 significantly associated variants that exceeded our Bonferroni-corrected significance threshold (SNPs < 1.15e−06; InDels < 7.88e−06; SVs < 5.92e−05) (Additional file 3: Table S19-S21). Strikingly, 99.77% (878 of 880), 92.22% (154 of 167), and 69.70% (23 of 33) of the significantly flat-fruit associated variants were located at the distal end (27.0–31.6 Mb) of Chr6 (Fig. 4a, b, Additional file 1: Fig. S12).

In the SNP-based GWAS output, the previously reported candidate SNP which is located at the fifth intron of *PpCAD1* [37] at position Chr6: 28,036,986 was the lead SNP in our results (Fig. 4a), supporting the reliability of our variants and analysis. The most significant association identified in the SV-based GWAS was a 1.67-Mb inversion from 27,959,880 bp to 29,634,101 bp at the “*S*” locus, covering the lead SNP. All the round-fruit peach accessions carried homozygous ancestral alleles (“*ss*”) and all the flat accessions (“*Ss*”) carried one ancestral allele and one derived allele harboring the inversion. We confirmed the heterozygous genotype by aligning RYP1 long contigs covering the flanking sequences around the breakpoints at both loci onto the RYP1 genome (Additional file 1: Fig. S13), and by performing Sanger sequencing of PCR-amplified flanking sequences at both breakpoints for each of the two alleles in the RYP1 accession (Fig. 4c). Considering the lack of inverted repeats at both breakpoints confirmed by the npINV program [63], it appears that this heterozygous 1.67-Mb inversion in flat-fruit accessions likely resulting from error-prone non-homologous end-joining (NHEJ) event (Fig. 4c), a process well-known to cause the deletion and/or insertion of nucleotides at the joint positions [64].

To further confirm that this region does indeed represent an inversion that is related to flat fruit phenotypes, we amplified the flanking sequences of the proximal breakpoint (Chr6: 27,959,880 bp) from the ancestral allele and the derived allele in the 136 accessions (including 99 round-fruit and 37 flat-fruit accessions). Supporting the inversion and its phenotypic association, all 37 of the flat-fruit accessions carried both the ancestral and derived alleles, whereas all 99 of the round-fruit accessions carried only the ancestral allele (Fig. 4d, Additional file 1: Fig. S14).

We next examined the linkage disequilibrium (LD) levels between SNPs positioned within or adjacent to the 1.67-Mb heterozygous inversion (Additional file 1: Fig. S15). The LD levels between distant SNPs were generally elevated in the flat-fruit accessions as compared such SNPs in the round-fruit accessions. We speculate that this increased LD in flat-fruit accessions probably results from suppression of recombination caused by the large inversion. This is plausible in light of reports about similar suppression of recombination in other species like great tits [65] and white-throated sparrow [66].

Analysis of LD levels between the 1.67-Mb heterozygous inversion and SNPs or InDels (Fig. 4e) supported that LD levels were higher around the inversion breakpoints and exhibited gradual decay in both directions from each of the breakpoints; a similar LD pattern has reported from a previous study of fruitfly [67]. We speculate that the high LD levels for SNPs and InDels in the significantly flat-fruit associated region may result from the “hitchhiking effect.” It is important to note that this flat-fruit-associated 1.67-Mb heterozygous inversion could not be identified if our GWAS had only used SNPs or InDels. That is, owing to the suppressed recombination rates and hitchhiking effects known to be caused by large SVs [68–70], the use of non-SV genetic variant data alone in our GWAS would almost certainly have missed the 1.67-Mb heterozygous inversion we discovered which can apparently explain the major agriculturally important fruit shape phenotype.

### The 1.67-Mb heterozygous inversion caused flat-fruit shape through upregulation of *PpOFP2* expression

**Fig. 5:**
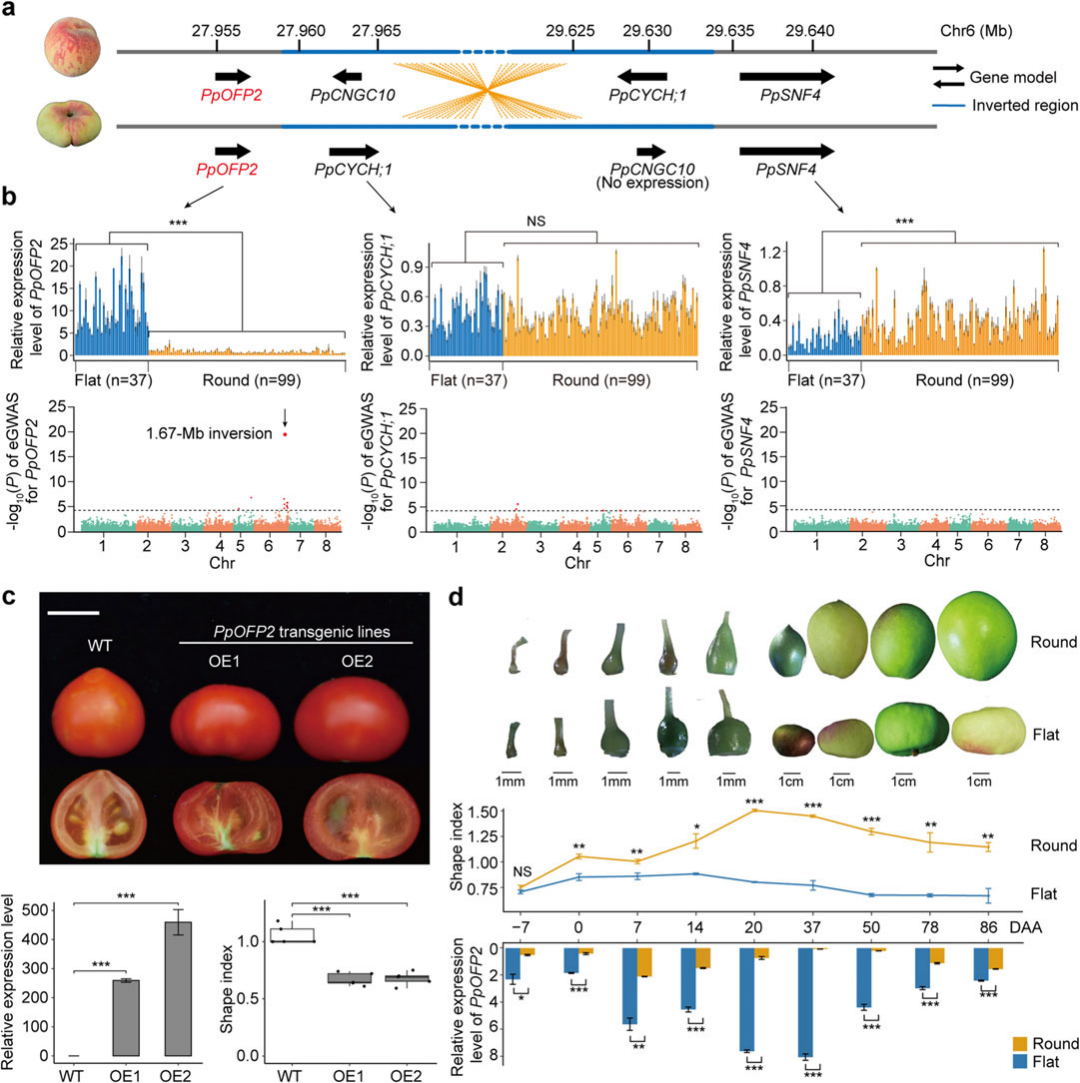
The 1.67-Mb heterozygous inversion caused flat fruit shape through upregulation of *PpOFP2* expression. **a** Genes proximal to the breakpoints of the 1.67-Mb inversion. **b** Relative expression levels of *PpOFP2* (top left), *PpCYCH;1* (top middle), and *PpSNF4* (top right) during early fruit development tested by qRT-PCR at 20 days after anthesis (DAA) in the 136 peach accessions, including 37 flat (blue bars) and 99 round (orange bars) accessions. ***P < 0.001 in Wilcoxon test. Genome-wide Manhattan plots of eGWAS based on SVs using relative expression levels of *PpOFP2* (bottom left), *PpCYCH;1* (bottom middle), and *PpSNF4* (bottom right) as phenotypes. **c** Fruit morphology (top), expression levels of *PpOFP2* (bottom left), and fruit shape index (bottom right) in wild type (WT) and two independent tomato transformants overexpressing *PpOFP2* (OE1 and OE2). Top plot, scale bar: 1 cm. The fruit shape index is calculated as the ratio of length to width. Bottom plots, *n* = 3, bar = SD, *** *P* < 0.001 in Wilcoxon test. **d** Fruit morphology (top), shape index (middle), and expression levels of *PpOFP2* (bottom) from early fruit development through near-mature fruit, in the representative round and flat accessions at 7 days before anthesis through 86 days after anthesis. Scale bars in top plot: 1 mm for the first five bars; 1 cm for the last four bars. For the middle and bottom plots, *n* = 3, bar = SD, NS (not significant) *P* > 0.05, *P < 0.05, **P < 0.01, ***P < 0.001 in Wilcoxon test

As the large heterozygous inversion being the casual variant for flat fruit shape, we first searched for disrupted genes at the corresponding boundary regions of the inversion; however, no genes were obviously disrupted by this large inversion. Large inversions often affect the expression of genes adjacent to the breakpoint owing to a variety of mechanisms [71–73]. Among the four breakpoint-adjacent genes (Fig. 5a), *PpOFP2* at ~ 3.12 kb of upstream of the proximal breakpoint was predicted to encode the protein containing an intact OVATE domain of the OFPs (Ovate Family Proteins) [74]; notably, the ortholog of *PpOFP2* (Additional file 1: Fig. S16) participated in determining fruit shape in tomato [75].

We used qRT-PCR to measure their expression levels in fruit tissue samples from the 136 genotyped accessions of our germplasm panel at 20 days after anthesis (DAA), a development stage that permits obvious detection of shape differentiation between round-fruit and flat-fruit accessions (Fig. 5d). *PpOFP2* and *PpSNF4* (located at ~ 2.75 kb of downstream of the distal breakpoint at 29,634,101 bp; Fig. 5a) showed differential expression between the round-fruit and flat-fruit peach groups (*P* < 0.001, Wilcoxon test) (Fig. 5b). The *PpCNGC10* gene was not expressed at the examined developmental stage, and the expression of the *PpCYCH;1* gene showed no difference between these two groups (Fig. 5b). Moreover, *PpOFP2* expression was significantly elevated in flat-fruit accessions, and an eGWAS (expression GWAS) analysis showed that only *PpOFP2* was significantly (*P* = 3.37e−20) associated with the 1.67-Mb inversion (Fig. 5b). All of these lines of evidence indicate that upregulation of *PpOFP2* expression is likely responsible for flat-fruit shape in peach. To further investigate the function of *PpOFP2*, we analyzed two independent transgenic tomato lines expressing *PpOFP2* driven by CaMV35S promoter. Phenotyping of the transgenic plants revealed that *PpOFP2* expression significantly reduces the length/width ratio of tomato fruit (Fig. 5c), thereby confirming the function of *PpOFP2* in promoting the development of flat fruits.

Differentiation between round and flat-fruit shape is known to be discernable at very early development stages after anthesis [39]. We collected samples from 9 different developmental stages, starting from 7 days before anthesis up to 86 days after anthesis for a flat (cv. Zhong You Pan 4) and a round (cv. Fuzador) peach accession, and measured *PpOFP2* expression (Fig. 5d). Throughout this developmental series, *PpOFP2* expression was consistently much higher in the flat peach than the round peach. Classic genetics research long ago established that flat-fruit shape in peach is controlled via a heterozygous dominant mode [31, 33, 35]. Our SV-data-guided identification of the 1.67-Mb inversion and our demonstration that this SV alters the expression of the causal gene *PpOFP2* collectively supports a very plausible explanation for this heterozygous mode: homozygosity for the derived allele (“*SS*” genotype) apparently results in a lethal dosage of *PpOFP2* expression that causes abortion of peach fruits during early development.

## Discussion

The high-quality RYP1 genome assembly generated in this study provides substantial improvements over the Lovell v2.0 reference genome in terms of longer contigs and completeness for repeat sequences (Table 1). This assembly enabled the identification of extensive numbers of hidden heterozygous SVs (9.68% of total genome size) in peach; this finding was surprising given the low heterozygosity of peach (0.22%). Our production of a comprehensive SV map for peach facilitates analyses to deepen understanding of peach population dynamics and selection. We detected extreme purifying selection against SVs during domestication and also identified a significant positive selection preference during peach improvement for SVs positioned at upstream regions (putative promoter regions) and intronic regions. Our SV map and identification of SV hotspots in peach will accelerate the utilization of this class of genetic variation in peach genetic improvement and will help drive additional hypothesis-driven research to understand the functional impacts of SVs on fruit and other agriculturally relevant phenotypes.

Previous studies of in human and other mammals have showed that large inversions modify gene expression via two major mechanisms: (i) directly disrupting regulatory elements adjacent to their breakpoints and (ii) rearranging regulatory elements positioned within or near to the inverted region [67, 76]. Recent findings showed that genomic rearrangements can rewire interactions of transcriptionally active enhancer elements and their proximal target genes, ultimately leading to human congenital limb disease and cancer [77–79]. We speculate that the mechanism underlying *PpOFP2’s* control of flat-fruit trait is very likely attributable to the rearrangement of regulatory elements that can activate expression positioned near inversion breakpoints. In this study, we indeed detected a significantly negative correlation (*r* = − 0.27, *P* < 0.001) between the expression patterns of *PpOFP2* and *PpSNF4* positioned near the proximal and distal breakpoints respectively in the 136 accessions (Additional file 1: Fig. S17). In the light of these results, we propose that activation of *PpOFP2* may be induced by the original regulatory element of *PpSNF4*, which was transferred downstream of *PpOFP2* by the inversion. Further investigation is warranted to accrue more evidence in support of this mechanistic hypothesis.

Our results provide a powerful illustration for how adopting an SV-level, whole-genome analytical approach can drive discovery in plant genomics and functional genomics research. It bears emphasis that our detection of the *PpOFP2* gene in peach as causal for flat-fruit shape resulted specifically from our ability to conduct an SV-based GWAS. A recently published study that included an analysis of flat fruit peach cultivars detected a related 1.7-Mb SV and reported a shortened silique phenotype transgenic Arabidopsis plants expressing the *PpOFP1* gene [80]. Besides confirming the impact of this locus on flat fruit shape, our study extended understandings for the fruit-related SV in at least three ways. First, we were able to harness our long-read-based RYP1 assembly to show that this SV is actually ~ 1.67 Mb in size, with the lack of any gaps accounting for this reduction. Second, we observed polymorphisms between two flat fruit cultivars (RYP1 and “124 Pan”) at both the proximal and distal breakpoints of the SV (compared in Additional file 1: Fig. S18). Finally, our study used the classic model fruit species tomato in its follow-up confirmatory analysis showing that an OVATE gene positioned near the proximal breakpoint of the SV does regulate the length/width ratio of tomato fruit.

It is undeniable that SNP-based GWAS represents a powerful experimental strategy for identifying genetic variations underlying plant traits [81–83]. However, there are limitations with the use of SNP data that can be overcome by adopting an SV-based GWAS approach. It is simply the case that some functionally impactful genomic variations are very large in scale (e.g., the heterozygous 1.67-Mb inversion of the present study), and many SVs driving tumorigenesis in various cancers [6, 8], and use of very high-resolution data like SNPs masks the ability to detect these larger-scale genomics phenomena. It is also worth noting that there is often suppression of recombination for large SVs [69, 70], which further exacerbates the difficulty of using SNP data to detect phenotypically impactful genetic variation: lacking normal recombination frequency, it is likely that a sufficient number of SNPs will prevent to enable researchers to detect causal mutations that are positioned within an SV. However, by using reads mapping and/or comparative genomic analyses to characterize SVs, it is often possible to immediately identify high-confidence candidate genes and variants for follow-up functional research [84].

## Conclusions

The roles of SVs on the evolution and regulation of plant genomes is a relatively unexplored area, so besides presenting both a high-quality peach genome assembly and a comprehensive SV map for a large number of genetically diverse accessions, our study in peach shows how SV data can be profitably used to gain basic functional insights and to facilitate genetic improvement programs.

## Methods

### Plant materials and growth conditions

All peach accessions used in this study were cultivated in the Beijing National Peach Germplasm Repository, China. Peach samples (leaves and fruits) were collected, frozen in liquid nitrogen, and stored at ultra-low temperature (− 80 °C) for later use. For *PpOPF2*-over-expression assays, the wild-type tomato (*Solanum lycopersicum* cv. Micro-Tom) and the transgenic lines were grown under greenhouse conditions with a 16 h-light and 8 h-dark photoperiod at 25 °C.

### Genome assembly and annotation

Extraction and purification of high molecular weight DNA was performed using a DNeasy Plant Maxi Kit (Qiagen, Germany). DNA concentration was measured using a NanoDrop (Thermo Fisher Scientific, USA) and Qubit 2.0 (Invitrogen, USA). One single-molecule real-time cell 8 M was run on the PacBio Sequel II platform, generating 7.25 million reads with a total length of 140.98 Gb (Additional file 2: Table S1). Illumina short-read data were obtained using Illumina NovaSeq platform, generating 235.81 million reads, with a total length of 35.37 Gb (Additional file 2: Table S1). The PacBio long-read data were de novo assembled into super contigs using Canu version 1.9 [40] and Highly Efficient Repeat Assembly (HERA) method [41]. The Illumina short-read data was used to polish the super contigs using Pilon [85]. The super contigs were then anchored into pseudo-chromosomes following the syntenic order of the Lovell v2.0 genome determined using MUMmer 4.0.0beta2 [45] (--mum -c 1000 -t 4).

The annotation of transposable elements was performed using RepeatMasker (http://www.repeatmasker.org). The repeat libraries included the RepBase-20170127 [86] and the de novo repeat library created using RepeatModeler. The pipeline for ab initio gene annotations included de novo gene predictions of the repeat-masked genome using AUGUSTUS [87] and SNAP [88], and evidence-based gene annotations using MAKER2 [89]. For de novo gene prediction, we used AUGUSTUS and SNAP trained on the homolog protein-coding genes collected from Swiss-Prot database for *Arabidopsis thaliana*, *Oryza sativa*, and *P. persica*. Transcript evidence included transcripts assembled from the RNA-Seq of different tissues (root, leaf, flower stages, and fruit; Additional file 2: Table S5) using HISAT and StringTie [90]. All the evidence was submitted to MAKER2, and the output of which was refined by searching against the InterPro database using InterProScan version 5.27–66.0 [91] to retain the genes with domain. Gene functional annotation was achieved using BLASTP (-evalue < 1e− 05) against the Swiss-Prot and NR databases. Gene Ontology terms of each gene were obtained from the corresponding InterPro entries. The pathways of each gene were assigned by BLASTP against the KEGG database, with an *E*-value cut-off of 1e−05.

### Evaluation of genome assembly

The flanking sequences of molecular markers from the high-density and multi-population consensus genetic linkage map for peach [42] were mapped against the RYP1 genome assembly using BLASTN. Only markers with unique alignment were used to evaluate the consistency between genome assembly and the genetic map. The Illumina short-read data were also used to evaluate the assembly accuracy and completeness using BWA-MEM version 0.7.17-r1188 [92]. Completeness of the genome assembly and gene annotations were assessed with a plant database of 1440 conserved plant genes (embryophyta_odb9) using BUSCO version 3.0.2 [93].

### Comparative genomics

Genome alignment among RYP1 and Lovell v2.0 was performed using NUCmer embedded in MUMmer with the parameters of “-mumreference -g 1000 -c 90 -l 40.” The delta-filter program was used to remove the mapping noise and determine the one-to-one alignment blocks with parameters “-r -q.” Gene duplications were analyzed with BLASTP with the parameters of “-evalue < 1e−0, -v 5, -b 5” for determination of the pairwise similarity between protein sequences of RYP1 and Lovell v2.0 genomes; the MCScanX package [94] was used for classification. OrthoFinder version 2.3.9 program [46] with the default parameters was used to create the orthogroups between proteomes of RYP1 and Lovell v2.0 genomes. To identify the presence/absence variations (PAVs) in the RYP1 genome, we divided the RYP1 genome into 500 bp overlapping windows with a step size of 100 bp. Each 500 bp window was then aligned against the Lovell v2.0 genome using BWA-MEM with the parameters of “-w 500 --M.” The sequences of the windows that failed to align with the Lovell v2.0 genome or those that aligned with less than 25% coverage were defined as RYP1-specific sequences. Overlapping windows that could not be aligned were merged. The Lovell-specific sequences were then identified following the same method.

### Heterozygous SV calling of RYP1 genome

In order to obtain comprehensive and high-quality SVs in the RYP1 genome, SVs were identified by combining three independent pipelines. First, PacBio contigs were aligned to the RYP1 genome using minimap2 version 2.17-r941 [95]. Four types of SVs (deletions, insertions, duplications, and inversions) were then identified using Assemblatron software (https://github.com/J35P312/Assemblatron). Second, the corrected Pacbio long reads were mapped to the RYP1 genome using pbmm2 (https://github.com/PacificBiosciences/pbmm2), followed by detection of the four SV types using pbsv (https://github.com/PacificBiosciences/pbsv). Last, to obtain more reliable detection outputs for inversions, the corrected Pacbio long reads were mapped to the RYP1 genome using minimap2, and then inversions were identified using npINV version 1.26 [63]. After detections, SVs were filtered by removing ones labeled as “BND” and ones that overlapped with assembly gaps for each output. The filtered SVs from all three pipelines were merged using SURVIVOR software [96] with default parameters.

### SNP, small InDel, and SV calling

Illumina resequencing data were generated for 149 peach accessions (Additional file 3: Table S13) with an average depth of 31.52×; among them, 148 accessions were newly sequenced. In addition, 37 accessions of *P. kansuensis* were newly sequenced to comprise the outgroup (Additional file 3: Table S13). The quality control for the raw re-sequencing data of the 186 accessions was performed using fastp version 0.20.1 [97] with default settings. For SNP and small InDel (≤ 30 bp) calling, Illumina short reads from the 186 accessions were aligned to the RYP1 genome using BWA-MEM; PCR duplicates were removed using Picard version 1.118 (http://broadinstitute.github.io/picard/). SNPs and InDels were identified using HaplotypeCaller in Genome Analysis Toolkit (GATK, version 4.1.5.0) [98] pipeline and then filtered following ref. [99].

Based on the same alignment files generated by BWA-MEM, we detected SVs in 186 re-sequenced accessions using the Manta program [100]; this program was selected for its high precision (low false discovery rate) and high recall performance, as demonstrated in a previously conducted evaluation of 10 highly cited short read structural variant calling programs [101]. We also confirmed the high accuracy of the Manta program for peach SV calling through a simulation study in comparison with the other two popular SV caller programs Delly [102] and IMR/DENOM [103] (Additional file 1: Fig. S19). For each accession, only SVs labeled with the flag “PASS” were retained. Finally, we merged all the SVs detected by Manta with the heterozygous SVs of RYP1 genome using SURVIVOR software with default parameters. The merged SVs were genotyped for the 186 accessions using Paragraph [104] that showed high sensitivity and accuracy of SV genotyping in the simulation study (Additional file 1: Fig. S19). SNPs, small InDels, and SVs were annotated using ANNOVAR [105].

### Validation of SNPs and SVs

To evaluate the accurate rate of SNP called based on resequencing data, we compared locations and genotypes of these SNP sites with those from our customized SNP genotyping array. A total of 74,692 common sites for 126 accessions (Additional file 3: Table S22) were overlapped and used to determined the validation rate. The validation rate for 126 accessions was 98.14–99.71% with a high total validation rate 99.18%, showing a low false positive rate (0.82%) of SNPs in this study, which was comparable with the validation rates reported from other studies about maize and sheep resequencing efforts [82, 106].

For SV, a total of 200 deletions and 100 insertions of 6 accessions including north, northwest, south, southwest China, Americas, and Europe (Additional file 3: Table S15) were selected to perform PCR-based validation. PCR was performed in 25 μl reaction volumes with 1 μl of genomic DNA (50 ng), 1 μl of forward and reverse forward primer (10 μM), and 22 μl of Golden Star T6 Super PCR mix (Beijing Tsingke Biotech Co., Beijing, China). PCR products were examined using 1.5% agarose-gel electrophoresis. The sizes of the amplified fragments were determined and used to infer the genotypes of deletions and insertions.

### Phylogenetic and population structure analysis

A total of 60,405 bi-allelic SNPs with a missing rate less than 50% and a minor allele frequency higher than 0.05 at fourfold degenerated sites were used for population analyses. A phylogenetic tree was built using FastTree v2.1.10 [107], and population structure was inferred using ADMIXTURE v1.3.0 [108] for each K value from 2 to 4, with 1000 bootstrap replicates.

### Determination of SV hotspots

We divided the RYP1 genome into 7985 non-overlapping 30-kb interval. We then mapped the total 27,734 SVs into the 7985 intervals. We assumed that the number of SVs mapped to each interval would follow a Poisson probability distribution if the SVs were distributed randomly across the genome, and we generated the expected Poisson distribution using the average number of SVs for each interval. The expected Poisson distribution was used to determine the criteria for SV hotspots. The intervals containing an empirical SV number equal to or higher than the 99th percentile of the expected Poisson probability distribution classified as SV hotspots. The gene ontology (GO) enrichment analysis was conducted using clusterProfiler [109]. The GO terms with the adjusted value smaller than 0.05 were considered as significant ones.

### Determination of NAHR-type SVs and segmental duplications

First, SVs with high coverage, more than 80%, of a variable number of tandem repeats (VNTRs) were excluded. Second, flanking sequences (± 100 bp) derived from both breakpoint junctions were aligned against each other to scan for blocks of extensive homology. SVs were classified as nonallelic homologous recombination (NAHR) if the homologous blocks had a minimum sequence identity of 85%, a minimum length of 20 bp for the identical sequences, a maximum offset of 20 bp between the homologous blocks, and also covered the breakpoints. The segmental duplications in RYP1 genome were identified using SEgmental Duplication Evaluation Framework (SEDEF) algorithm [110].

### Principle component analysis

PCA was performed for the SNPs and SVs using smartPCA program embedded in Eigensoft package version 7.2.1 [111].

### Distribution of fitness effects of SVs

The unfolded site frequency spectrum (SFS) of sSNPs, nSNPs, DELs, INSs, and DUPs for landraces and modern cultivars were computed using *P. kansuensis* as the outgroup. The program polyDFE (v2.0) [112] was used to estimate the distribution of fitness effects for nSNPs, DELs, INSs, DUPs, and INVs with the parameters “-m A -e –b.” These analyses based on the unfolded SFS and sSNPs were used as neutral references. As polyDFE cannot deal with missing data, and also to decrease the parameters to be estimated, we projected the SFS of both landraces and modern cultivars to a sample size of 20. The standard deviation was estimated by analyzing 100 bootstrap replicates of SFS.

### Genetic nucleotide diversity

Genetic nucleotide diversity (*θ*π, the average number of nucleotide differences per site between two randomly chosen DNA sequences from the population) were calculated using VCFtools version 0.1.16 [113].

### Identification of highly divergent SVs between landraces and modern cultivars

To identify highly divergent SVs between landraces and modern cultivars, the numbers of accessions with or without genotypes for each SV were compared using Fisher’s exact test. The raw *P* values were adjusted for multiple tests using FDR [114]. SVs were considered to be significantly divergent when the FDR value < 0.01.

### GWAS and eGWAS

SNPs, small InDels, and SVs were imputed using Beagle v4.1 [115] with default parameters. GWAS analysis was conducted using 869,345 SNPs, 191,279 small InDels (≤ 30 bp), and 16,883 SVs (MAF ≥ 0.05) using the Efficient Mixed-Model Association eXpedited (EMMAX) algorithm [116]. In order to detect expression-associated SVs for the three genes proximal to the breakpoints of the 1.67-Mb inversion, GWAS was performed using EMMAX algorithm with the expression pattern of the three genes in the population of 136 accessions as the phenotypes.

### Validation and quantification of gene expression

The expressions of *PpOFP2*, *PpCYCH;1*, *PpCNGC10*, and *PpSNF4* were quantified in 136 peach accessions (Additional file 3: Table S13) using qRT-PCR. Total RNA was extracted from ovary tissues at 20 days after anthesis (DAA) using a RNAprep Pure Plant Plus Kit (Polysaccharides & Polyphenolics-rich) (TIANGEN, China). First-strand cDNA was synthesized using a PrimeScript™ RT Reagent Kit with gDNA Eraser (Takara, Japan). Quantitative PCR was performed using SYBR Green Real-time Master Mix (Takara, Japan), following the manufacturer’s instructions, on a StepOnePlus™ Real-Time PCR System (Applied Biosystems, USA). cDNA transcript levels were normalized to that of the reference gene *PpG0000030306.01* (*actin*), as described in a previous study [117], using the 2^-ΔΔCT^ method [118]. Primers (Additional file 3: Table S23) were designed to span an intron in order to avoid amplification of genomic DNA. PCR reactions were performed in triplicate for each biological replication.

### Validation and genotyping of the 1.67-Mb heterozygous inversion

Genomic DNAs were extracted from the young leaves of 136 peach accessions (Additional file 3: Table S13) using a Hi-DNAsecure Plant Kit (TIANGEN, China). Four primer pairs (P1, P5, P6, and P7; Additional file 3: Table S23) were designed to detect the flanking sequence of both breakpoints of both alleles for the 1.67-Mb inversion genotypes of the RYP1 cultivar. Another four primer pairs (P1, P2, P3, and P4; Additional file 3: Table S23) were designed to detect the flanking sequences of the proximal breakpoint of both alleles for the 1.67-Mb inversion genotypes of the 136 peach accessions. PCR reactions were carried out using Premix Taq™ (Ex Taq™) (Takara, Japan) in a volume of 20 μl, containing 20 ng of peach genomic DNA. PCR was carried out under the following conditions: 2 min at 95 °C, 30 cycles of 30 s at 95 °C, 30 s at 60 °C, and 90 s at 72 °C, followed by a 5 min extension at 72 °C. PCR products were examined using agarose-gel electrophoresis.

### Generation of transgenic tomato plants

Primer pairs OFP2-F/R (Additional file 3: Table S23), which added *Xba*I and *Kpn*I sites to the 5′ and 3′ ends, respectively, were designed to amplify the intact coding sequence of *PpOFP2* using cDNA templates from RYP1 fruits at 30 DAA. PCR products were then digested with *Xba*I and *Kpn*I and cloned into the binary pCAMBIA1300 vector. The recombinant plasmid was transformed into *Agrobacterium tumefaciens* EHA105 competent cells via electroporation prior to subsequent plant transformation. The protocol for tomato (Micro-Tom) transformation was taken from a previous study [119]. The tomato wild type and transgenic lines were grown in a growth chamber with a 16-h-light and 8-h-dark photoperiod at 25 °C. The transgenic lines were screened using hygromycin and identified by qRT-PCR analysis using the *RPL2* gene in tomato [120] as the control (Additional file 3: Table S23).

## Supplementary Information

**Additional file 1: Supplementary Figs. S1–S19.** This file contains the supplementary figures referenced in the main text. **Fig. S1.** Overview of the pipeline used for the RYP1 genome assembly. **Fig. S2.** Consistency of physical and genetic maps. **Fig. S3.** The RYP1 and Lovell v2.0 genome assemblies evaluated by LTR Assembly Index (LAI). **Fig. S4.** Colinearity between the RYP1 genome assembly and the Lovell v2.0 reference genome. **Fig. S5.** Kyoto Encyclopedia of Genes and Genomes (KEGG) pathway analysis of Lovell v2.0 (a) and RYP1 (b) specific and expansion genes, and the phylogenetic tree of expansion genes related to the fructose and mannose metabolism (c). **Fig. S6.** Phylogenetic tree and model-based clustering analysis of the population (149 *P. persica* accessions and 37 wild relatives, *P. kansuensis*, as the outgroup) constructed using 60,405 SNPs at fourfold degenerate sites (Missing rate < 50%, Minor allele frequency > 0.05). **Fig. S7.** SV genotyping summary for 149 peach accessions. **Fig. S8.** Principal component analyses (PCA) of 149 accessions including 41 landraces and 108 modern cultivars based on SNPs (a) and SVs (b). **Fig. S9.** Segmental duplications and formation mechanisms of SVs within hotspot and non-hotspot intervals. **Fig. S10.** Heterozygosity ratio based on SVs in landraces (*n* = 41) compared to that of modern cultivar (*n* = 108) populations. **Fig. S11.** Number of shared SNPs (a) and SVs (b) between landraces and modern cultivars. **Fig. S12.** GWAS analysis for fruit shape (round/flat) based on genome-wide small InDels (≤30 bp). **Fig. S13.** The alignment of the RYP1 contigs against the RYP1 genome. **Fig. S14.** Agarose-gel electrophoresis of PCR products from the 136 peach accessions, including 37 flat and 99 round accessions. **Fig. S15.** The LD heatmap of round (*n* = 99) (a) and flat peach (*n* = 37) (b) groups on Chr6: 27.0–31.6 Mb. **Fig. S16.** Multiple protein sequence alignment (a) and a neighbor-joining phylogenetic tree (b) of PpOFP2 altogether with other 19 OFPs from Arabidopsis (*At*), rice (*Os*), and tomato (*Sl*) based on their coding sequences. **Fig. S17.** Correlation of relative expression level of *PpOFP2* and *PpSNF4* gene in 136 peach accessions. **Fig. S18.** Comparison of polymorphisms flanking the breakpoints of the heterozygous inversion between RYP1 and ‘124 Pan’ peach cultivars. **Fig. S19.** Evaluation of SV caller programs using simulated short-read data.

**Additional file 2: Supplementary Tables S1–S7, S10–S12, S14.** This file contains the supplementary tables referenced in the main text. **Table S1.** Summary of sequencing data. **Table S2.** Number and size of each chromosome (Chr) and unanchored contigs for the RYP1 genome. **Table S3.** Statistics for contigs/scaffolds that could not be anchored onto chromosomes in the RYP1/Lovell v2.0 genome assemblies. **Table S4.** The genome assembly and annotation completeness of the RYP1 and Lovell v2.0 genomes assessed by BUSCO. **Table S5.** Summary of statistics for transcriptional data from RNA sequencing (RNA-seq) analysis of different tissues for gene model prediction. **Table S6.** Annotation statistics of predicted protein-coding genes for the RYP1 genome. **Table S7.** Summary of repeat elements identified in the RYP1 genome and the Lovell v2.0 genome. **Table S10.** Summary statistics of orthogroups between the RYP1 and Lovell v2.0 genomes. **Table S11.** Summary of duplicated genes in the RYP1 and the Lovell v2.0 genomes. **Table S12.** Summary statistics of heterozygous variants (SNPs, InDels and SVs) in the RYP1 genome. **Table S14.** Number and length of different types of SVs in peach.

**Additional file 3: Supplementary Tables S8–S9, S13, S15–S23.** This file contains the supplementary tables referenced in the main text. **Table S8.** Information about Lovell and Rui You Pan1 (RYP1). **Table S9.** Specific PAVs for the RYP1 and Lovell v2.0 genomes. **Table S13.** Basic information and statistics of 186 re-sequenced peach accessions (including 149 *P. persica* and 37 *P. kansuensis*) used in this study. **Table S15.** PCR validation of randomly selected SVs. **Table S16.** Enriched gene ontology (GO) terms with coding or upstream (< 1 kb) regions (putative promoters) affected by SVs. **Table S17.** The SVs occurred in gene cluster of receptor-like protein kinase gene *LRK10* in the three consecutive SV hotspots. **Table S18.** Highly divergent SVs during peach improvement. **Table S19.** SNPs significantly associated with flat fruit shape. **Table S20.** InDels significantly associated with flat fruit shape. **Table S21.** SVs significantly associated with flat fruit shape. **Table S22.** Summary information for SNPs of 126 accessions identified in this study with genotyped SNPs from customized SNP genotyping array. **Table S23.** PCR or qRT-PCR primers used in this study.

**Additional file 4.** Review history.

## Data Availability

The raw resequencing data and transcriptome data have now been deposited in Sequence Read Archive (https://www.ncbi.nlm.nih.gov/sra/) of National Center for Biotechnology Information (NCBI) under BioProjects PRJNA663114 [121] and PRJNA664002 [122], respectively. This whole genome shotgun project has been deposited at GenBank under the accession JACYOW000000000 [123]. The raw PacBio data was available in the NCBI Sequence Read Archive under BioProject PRJNA663129 [124]. The SNP and SV data in Variant Call Format, full pipeline strategy, and associated scripts can be freely and openly accessed on figshare (10.6084/m9.figshare.12937340.v1) [125].
