# Supplementary material for: Genome structure variation analyses of peach reveal population dynamics and a 1.67 Mb causal inversion for fruit shape
Source: Genome Biol. 2021 Jan 5;22:13. doi: 10.1186/s13059-020-02239-1 (PMC7784018; doi:10.1186/s13059-020-02239-1)
Supplement: Supplementary file 1 — Additional file 1: Supplementary Figs. S1–S19. This file contains the supplementary figures referenced in the main text. Fig. S1. Overview of the pipeline used for the RYP1 genome assembly. Fig. S2. Consistency of physical and genetic maps. Fig. S3. The RYP1 and Lovell v2.0 genome assemblies evaluated by LTR Assembly Index (LAI). Fig. S4. Colinearity between the RYP1 genome assembly and the Lovell v2.0 reference genome. Fig. S5. Kyoto Encyclopedia of Genes and Genomes (KEGG) pathway analysis of Lovell v2.0 (a) and RYP1 (b) specific and expansion genes, and the phylogenetic tree of expansion genes related to the fructose and mannose metabolism (c). Fig. S6. Phylogenetic tree and model-based clustering analysis of the population (149 P. persica accessions and 37 wild relatives, P. kansuensis, as the outgroup) constructed using 60,405 SNPs at fourfold degenerate sites (Missing rate < 50%, Minor allele frequency > 0.05). Fig. S7. SV genotyping summary for 149 peach accessions. Fig. S8. Principal component analyses (PCA) of 149 accessions including 41 landraces and 108 modern cultivars based on SNPs (a) and SVs (b). Fig. S9. Segmental duplications and formation mechanisms of SVs within hotspot and non-hotspot intervals. Fig. S10. Heterozygosity ratio based on SVs in landraces (n = 41) compared to that of modern cultivar (n = 108) populations. Fig. S11. Number of shared SNPs (a) and SVs (b) between landraces and modern cultivars. Fig. S12. GWAS analysis for fruit shape (round/flat) based on genome-wide small InDels (≤30 bp). Fig. S13. The alignment of the RYP1 contigs against the RYP1 genome. Fig. S14. Agarose-gel electrophoresis of PCR products from the 136 peach accessions, including 37 flat and 99 round accessions. Fig. S15. The LD heatmap of round (n = 99) (a) and flat peach (n = 37) (b) groups on Chr6: 27.0–31.6 Mb. Fig. S16. Multiple protein sequence alignment (a) and a neighbor-joining phylogenetic tree (b) of PpOFP2 altogether with other 19 OFPs from Arabidopsis ( [file 13059_2020_2239_MOESM1_ESM.docx]

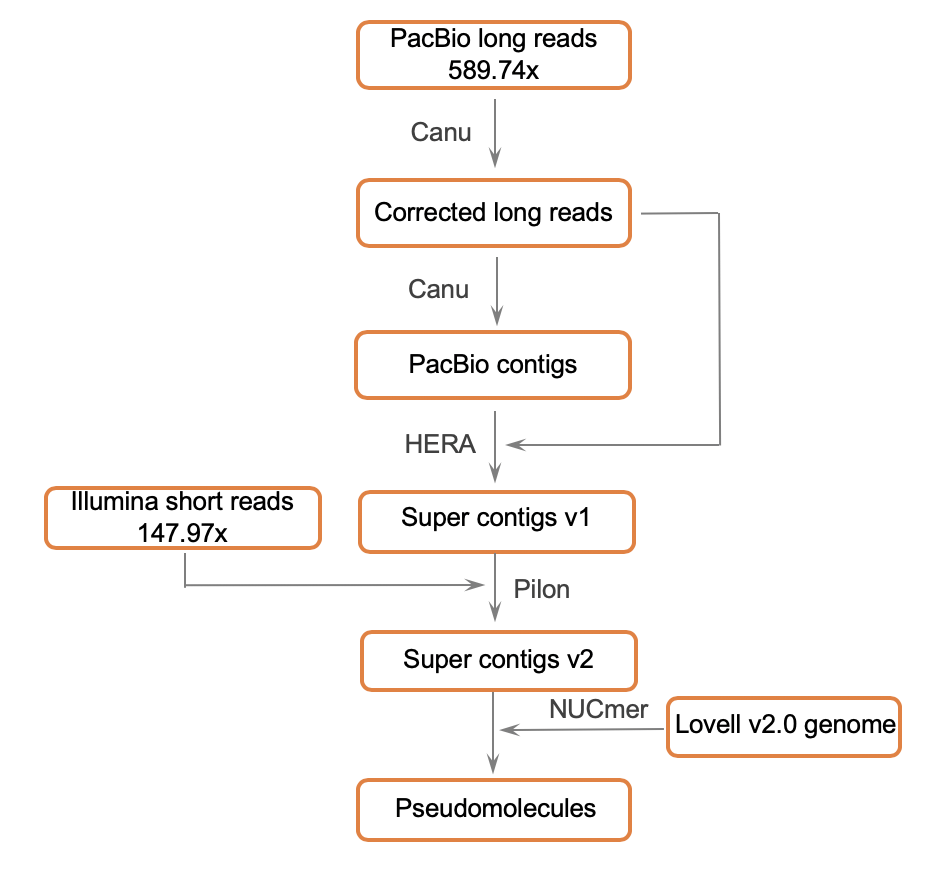


**Fig S1 Overview of** **the pipeline used for the RYP1 genome assembly.** The PacBio long reads for RYP1 were corrected and assembled into PacBio contigs using the Canu pipeline (version 1.8). The PacBio contigs and corrected long reads were assembled into super contigs v1 by the HERA algorithm. After correction of all these super contigs with Illumina short reads of RYP1 using the Pilon program, the super contigs v2 were then anchored into chromosome-level pseudomolecules according to their alignment with the Lovell v2.0 reference genome by the NUCmer program.

**Fig S2** **Consistency of physical and genetic maps.** Comparison between the positions of 2,257 molecular markers in the genetic maps and their positions on the RYP1 genome, with a high proportion (93.09%) located at their expected positions. Each marker is depicted as a dot on the plot.


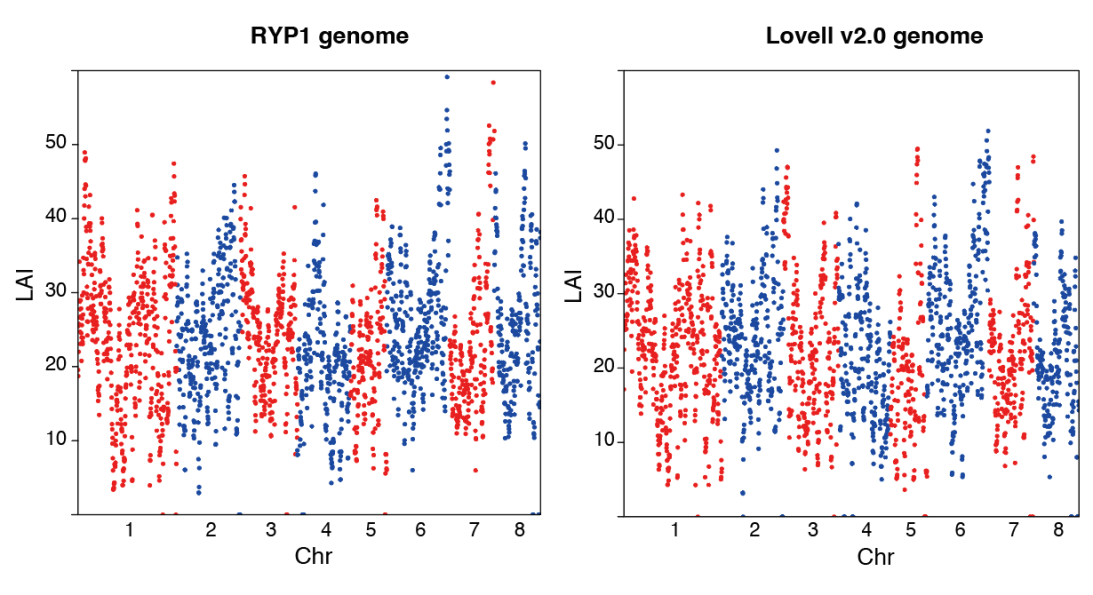


**Fig S3 The RYP1 and Lovell v2.0 genome assemblies evaluated by LTR Assembly Index (LAI).** Most regions in the RYP1 genome showed higher LAI values than those in the Lovell v2.0 genome, indicating RYP1 had better assembly continuity.


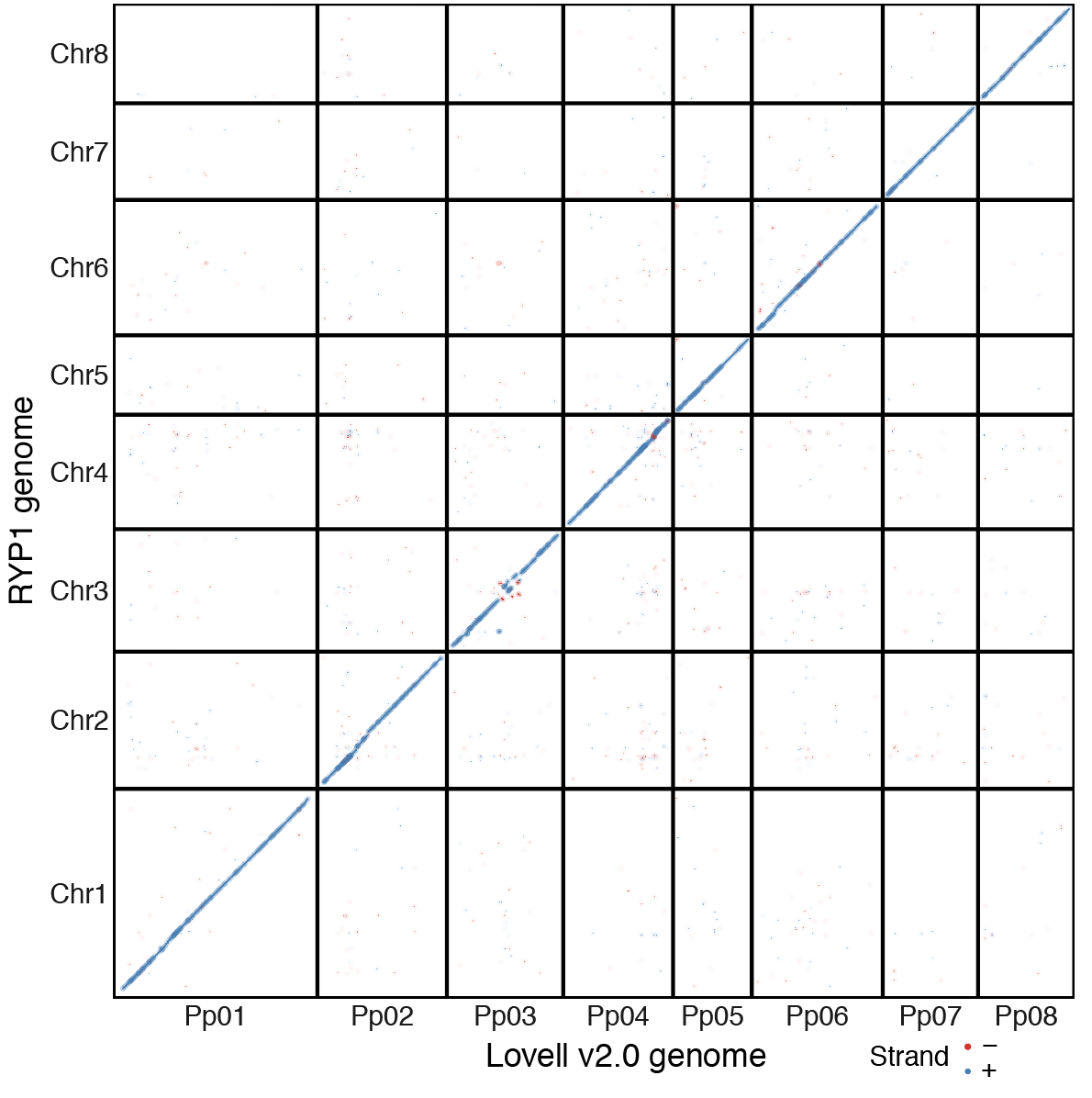


**Fig S4** **Colinearity between the RYP1 genome assembly and the Lovell v2.0 reference genome.** Each dot indicates an aligned region with a length of at least 10 kb.


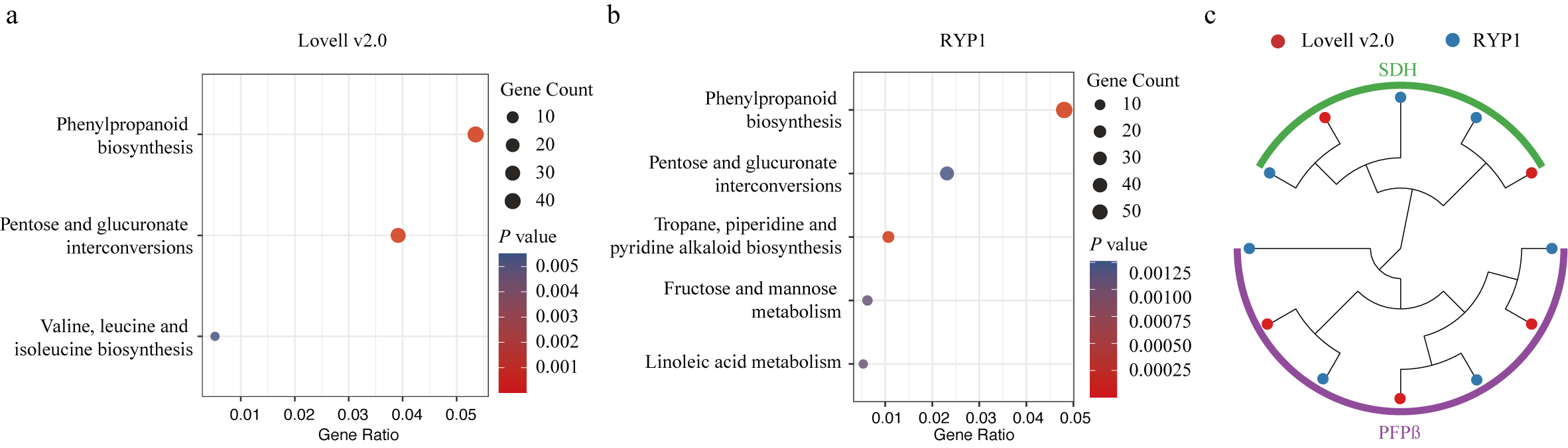


**Fig S5 Kyoto Encyclopedia of Genes and Genomes (KEGG) pathway analysis of Lovell v2.0 (a) and RYP1 (b) specific and expansion genes, and the phylogenetic tree of expansion genes related to the fructose and mannose metabolism (c).** X-axis represents the ratio of the enriched gene number among the total analyzed genes. The size of the round nodes is in proportion to the number of genes significantly enriched in each pathway. Color of round node represents the *P* value of significant KEGG pathway. Sorbitol dehydrogenase (*SDH*). Pyrophosphate fructose-6-phosphate 1-phosphotransferase subunit beta (*PFPβ*).


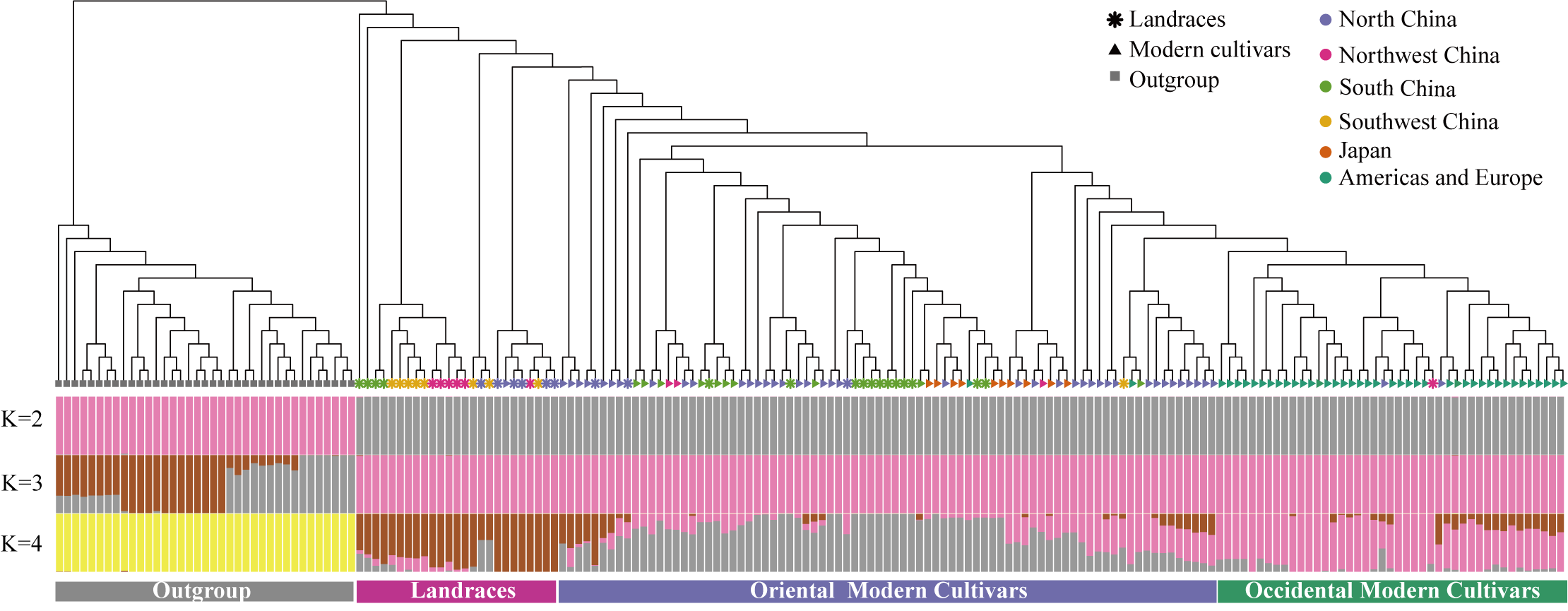


**Fig S6 Phylogenetic tree and model-based clustering analysis of the population (149 *P. persica* accessions and 37 wild relatives, *P. kansuensis*, as the outgroup) constructed using 60,405 SNPs at fourfold degenerate sites (Missing rate < 50%, Minor allele frequency > 0.05).**


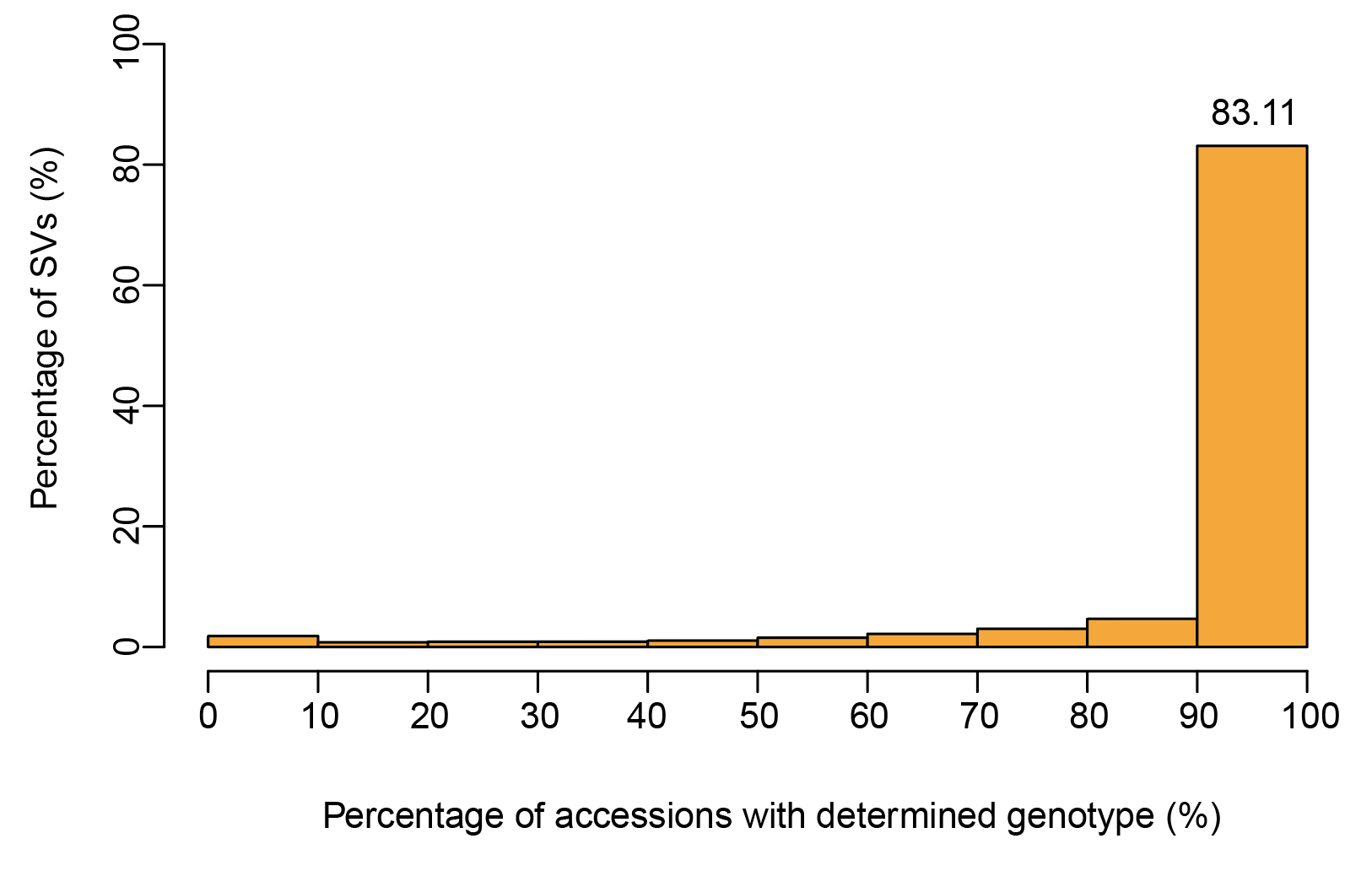


**Fig S7 SV genotyping summary for 149 peach accessions.**

**Fig S8** **Principal component analyses (PCA) of 149 accessions including 41 landraces and 108 modern cultivars based on SNPs (a) and SVs (b).** The SNPs and SVs with MAF ≤ 0.05 and ≥ 50% missing data were removed.


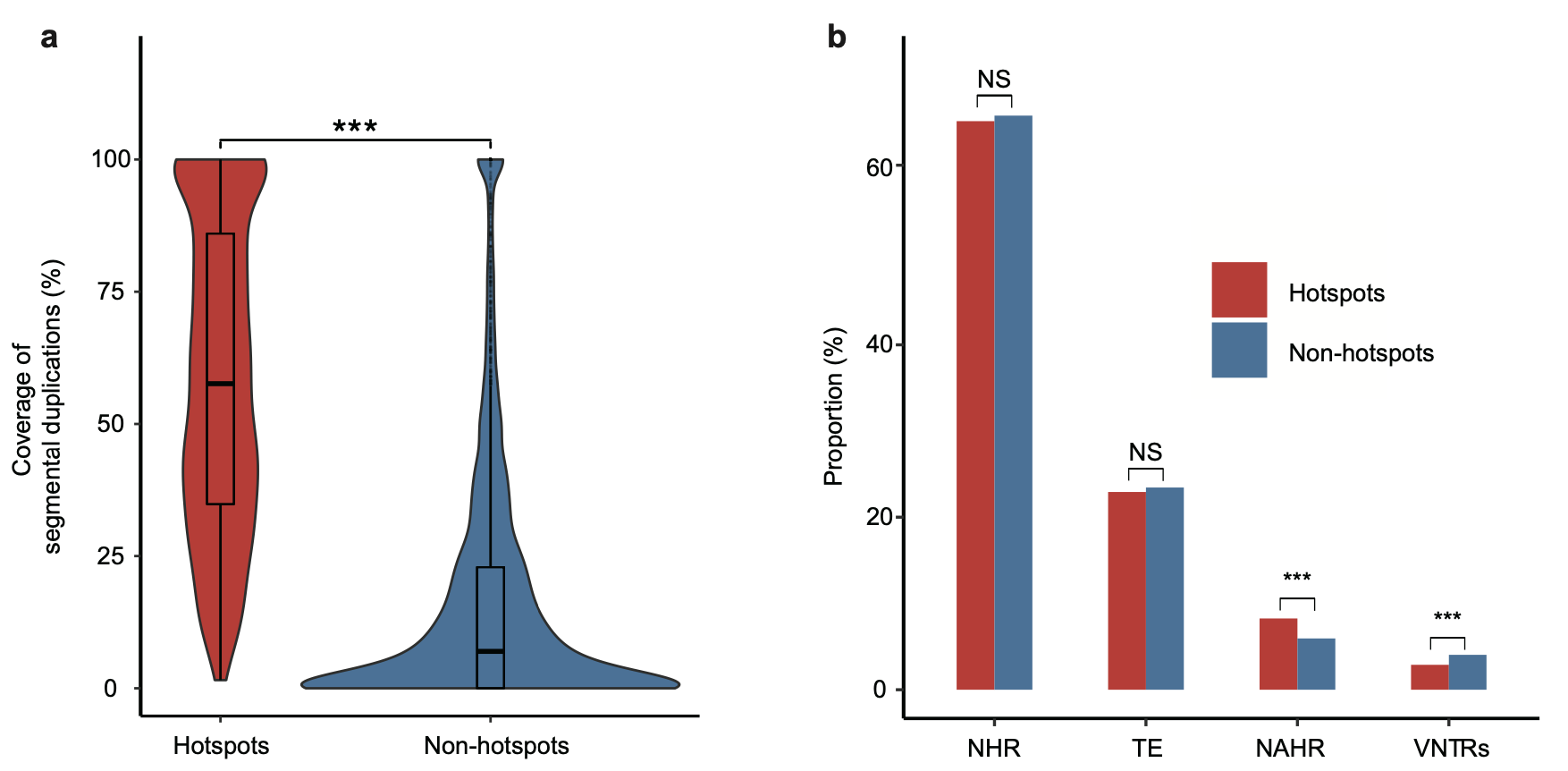


**Fig S9** **Segmental duplications and formation mechanisms of SVs within hotspot and non-hotspot intervals.** (**a**) Violin and box plot comparing the coverages of segmental duplications within hotspot and non-hotspot intervals. (**b**) Bar plot showing the co-incidence of NHR (nonhomologous recombination), TE (transposon element-related), NAHR (non-allelic homologous recombination), and VNTRs (variable number of tandem repeats-related) formation mechanisms of SVs within hotspot and non-hotspot regions. The proportions of SVs attributed to individual mechanisms are plotted. NS (Not significant) *P* > 0.05, *** *P* < 0.001 in Fisher’s exact test.


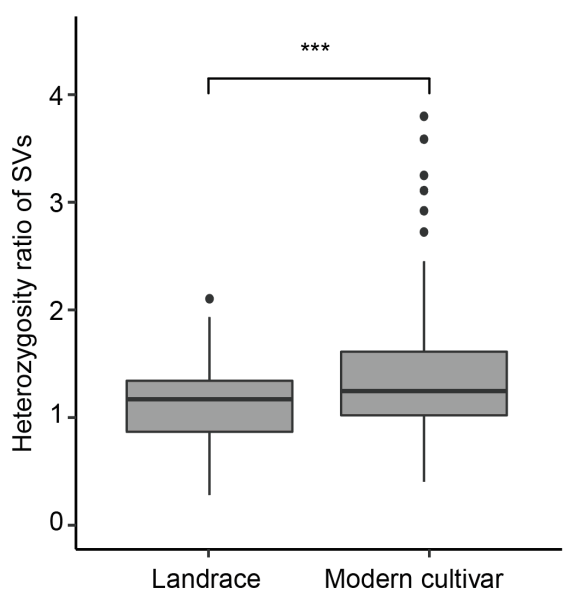


**Fig S10** **Heterozygosity ratio based on SVs in landraces (n = 41) compared to that of modern cultivar (n = 108) populations.** *** *P* < 0.001, calculated by Wilcox test. In box plot, central line: median, bounds of box: 25^th^ and 75^th^ percentiles, whiskers: 1.5 * IQR (IQR: the interquartile range between the 25^th^ and 75^th^ percentile).


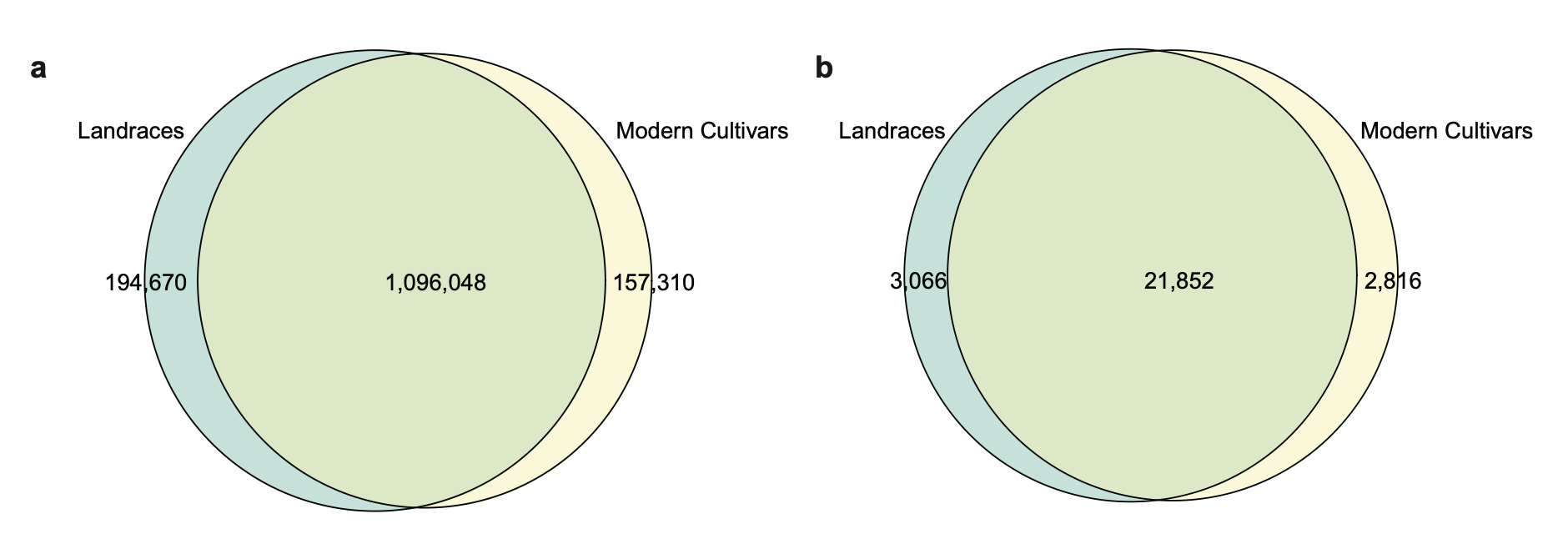


**Fig S11 Number of shared SNPs (a) and SVs (b) between landraces and modern cultivars.** The proportions for shared SVs (among 21,852 total common SVs, 87.70% for landraces and 88.58% for modern cultivars), and SNP-based analysis (84.92% for landraces and 87.45% modern cultivars). The total number of SNPs and SVs are 1,448,028 and 27,734, respectively.


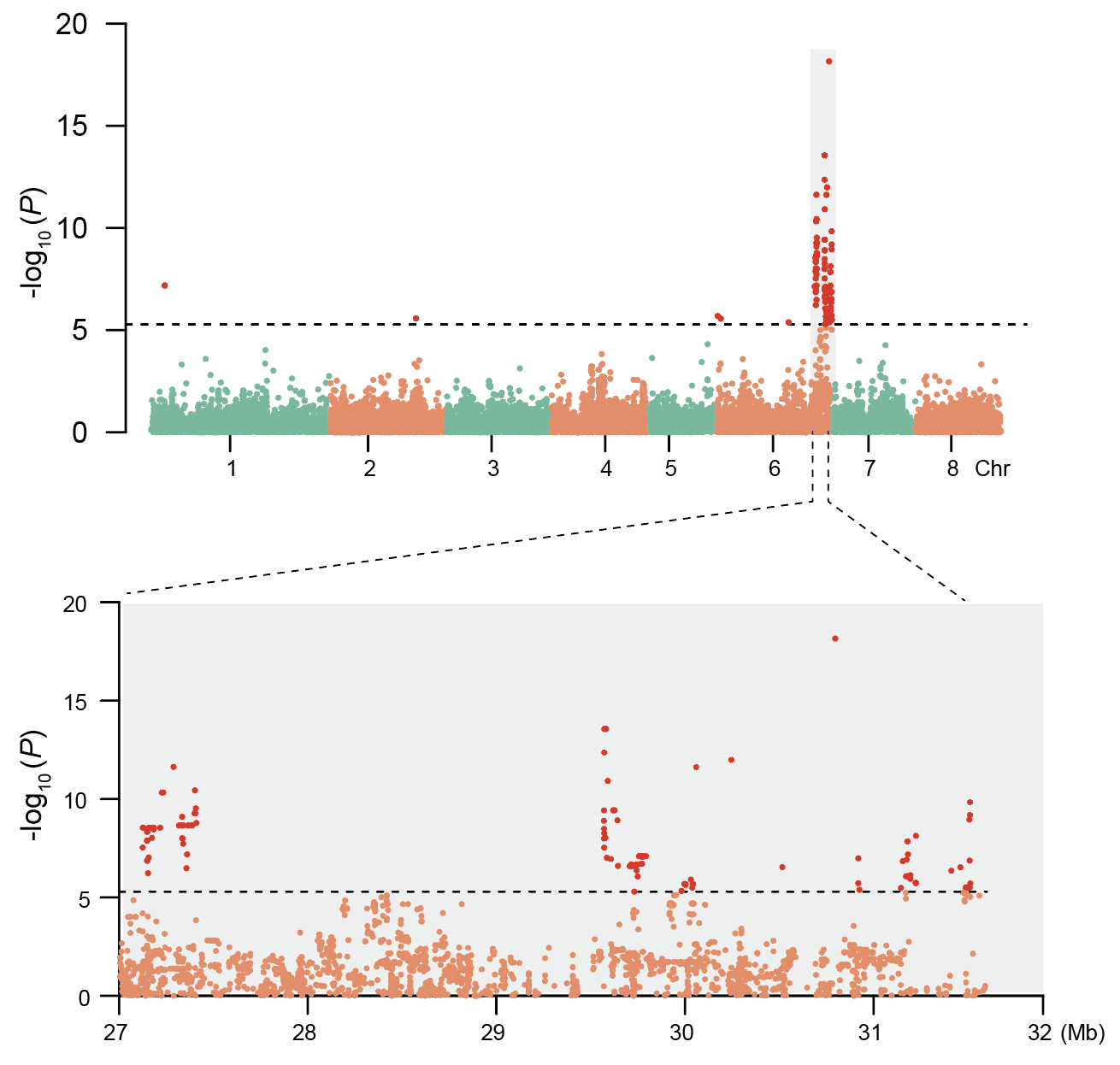


**Fig S12 GWAS analysis for fruit shape (round/flat) based on genome-wide small InDels (≤30bp).** Genome-wide (top) and regional (bottom) Manhattan plots of GWAS analysis. Regional plots show the most significant region of chr6. Each dot represents an InDel. Horizontal dashed black lines correspond to the Bonferroni-corrected significance threshold (5.10).


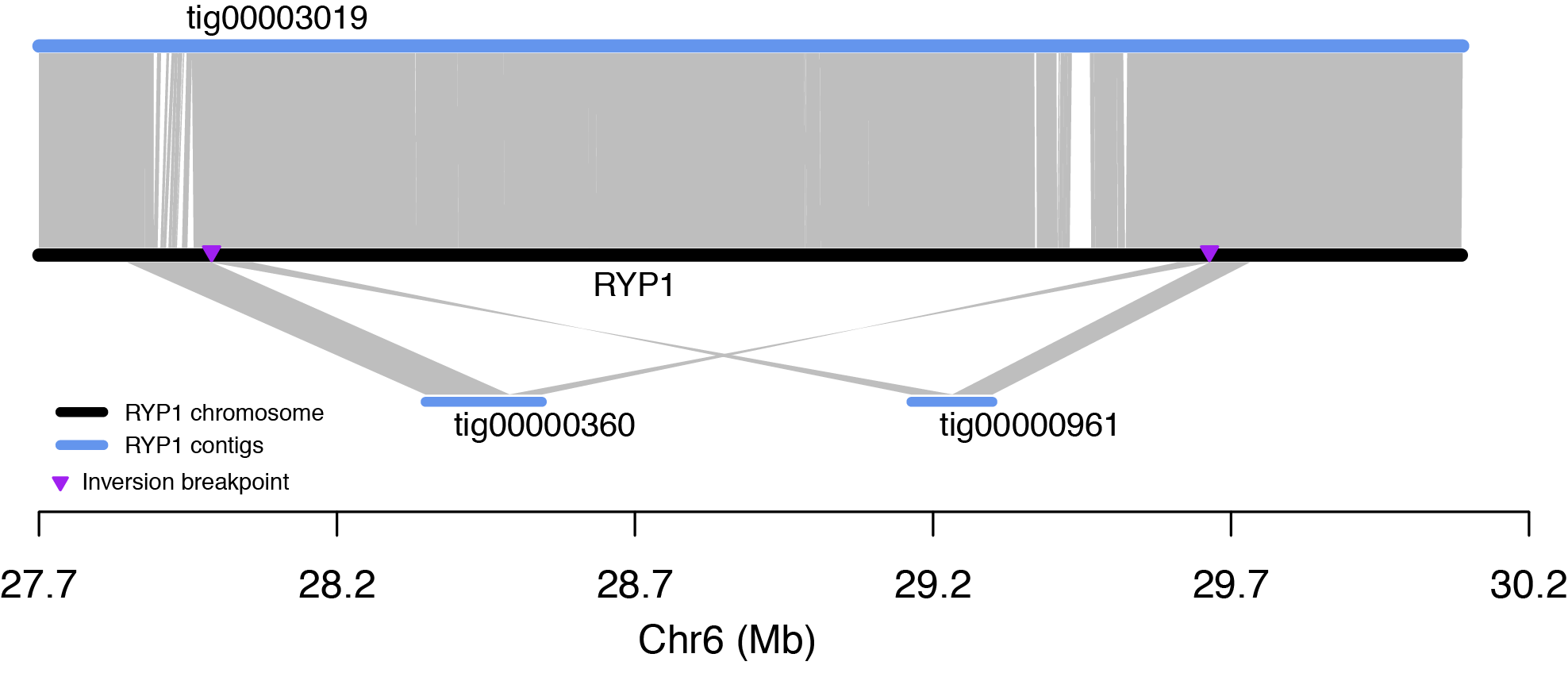


**Fig S13 The alignment of the RYP1 contigs against the RYP1 genome.** The grey lines indicate the aligned regions. The blue horizontal lines show the RYP1 contigs. The middle black line indicates the Lovell v2.0 genome. The purple triangle indicates the breakpoints of the 1.67-Mb inversion.


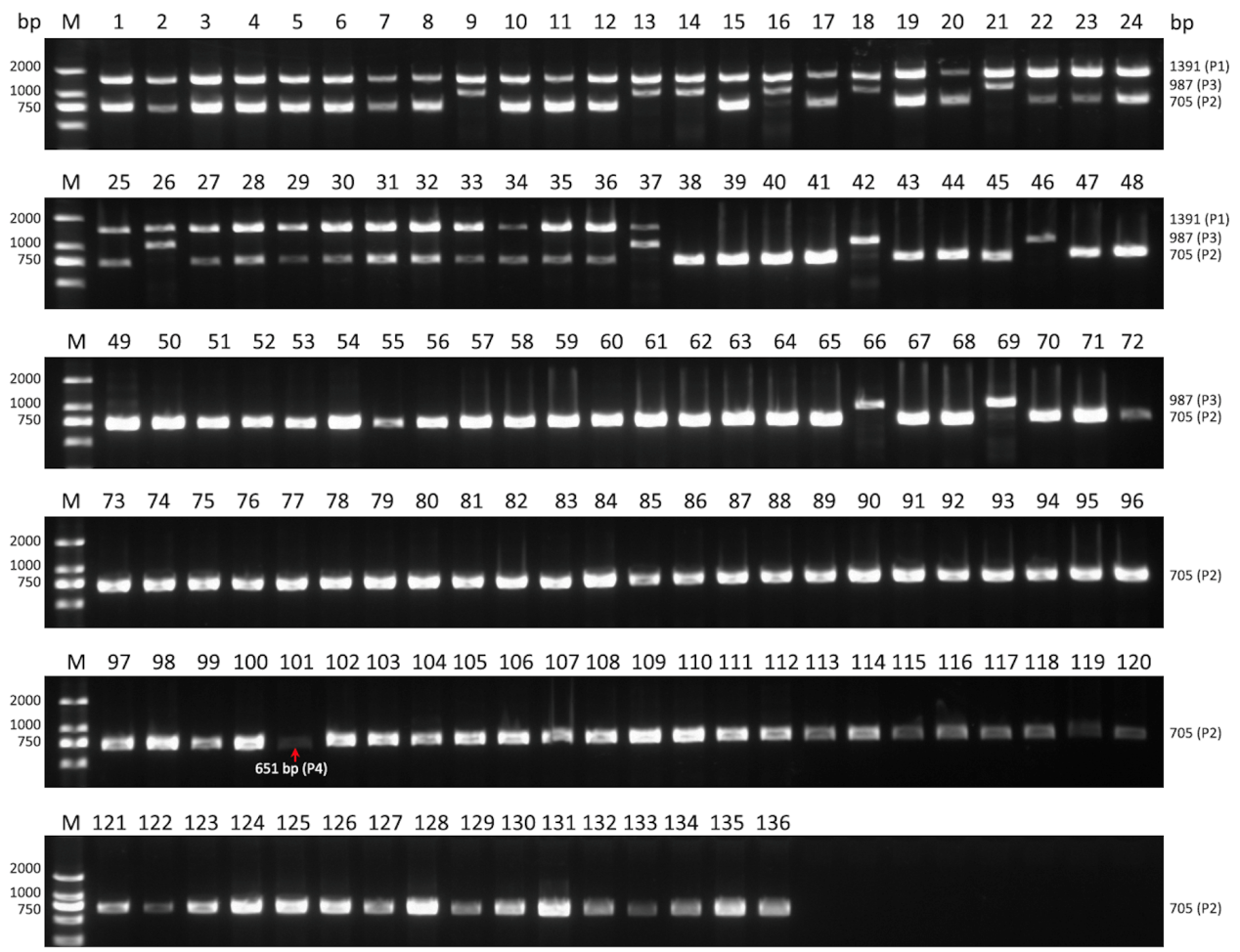


**Fig S14 Agarose-gel electrophoresis of PCR products from the 136 peach accessions, including 37 flat and 99 round accessions.** To validate the association of the 1.67-Mb heterozygous inversion with flat shape, multiple primers were designed. Primer pair P1 was used to amplify the flanking sequence of the proximal breakpoint of the derived allele, while P2, P3, and P4 were used to amplify the proximal breakpoint of the ancestral allele. Amplification with P1 gave a fragment with the expected size of 1,391 bp in the flat peach accessions (lane 1 to 37), but failed in the round ones (lane 38 to 136). P2, P3, and P4 were used as positive controls which produced 705 bp, 987 bp, and 651 bp products, respectively. M indicates DNA ladders. Information for individual accessions is listed in Additional file 2: Table S11.

**Fig S15** **The LD heatmap of round (n = 99) (a) and flat peach (n = 37) (b) groups on Chr6: 27.0–31.6 Mb.** Inversion breakpoints are depicted as vertical dashed blue lines.

a

b

**Fig S16** **Multiple protein sequence alignment (a) and a neighbor-joining phylogenetic tree (b) of PpOFP2 altogether with other 19 OFPs from Arabidopsis (*At*), rice (*Os*), and tomato (*Sl*) based on their coding sequences.** In (**a**) black and gray shading show amino acids that are identical or similar, respectively; the black points and asterisks indicate positions with more than 50% and 100% conserved amino acids, respectively, in the alignment; the conserved OVATE domain in the C-terminus is underlined with red lines. In (**b**) phylogenetic analysis was conducted using MEGA 5 and statistical support for each node was calculated using 2,000 bootstraps.


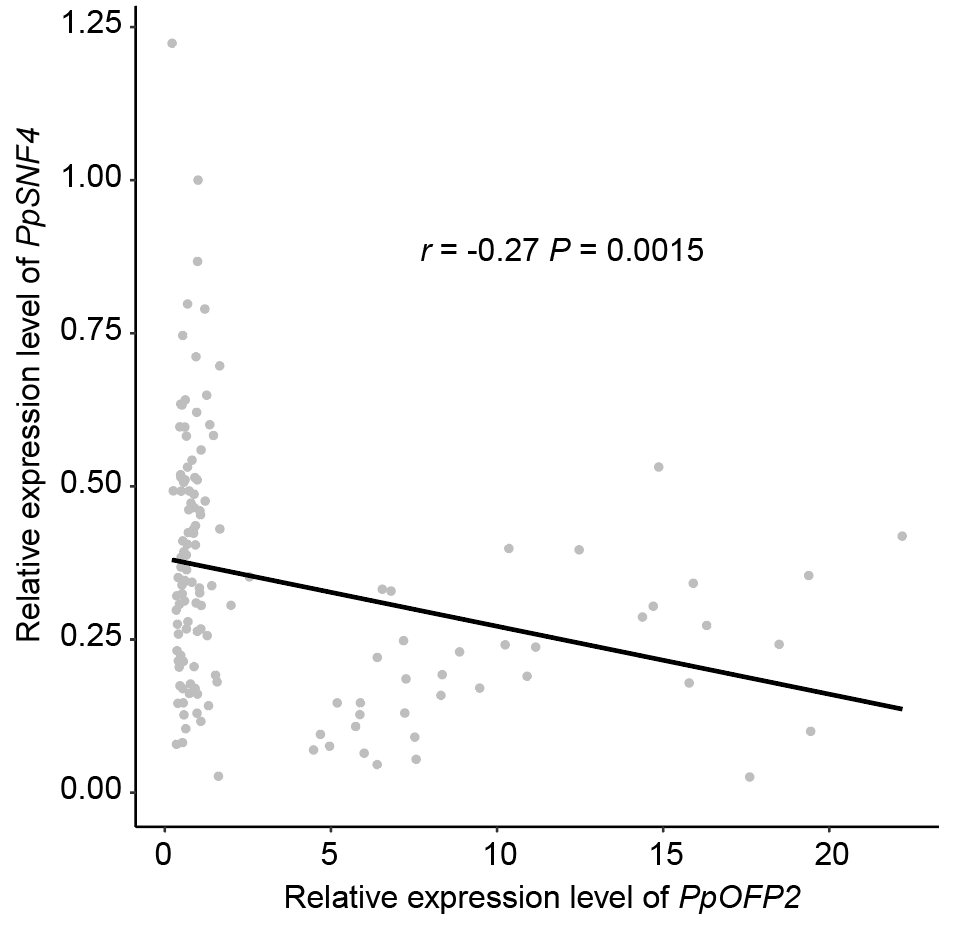


**Fig S17 Correlation of relative expression level of *PpOFP2* and *PpSNF4* gene in 136 peach accessions.**


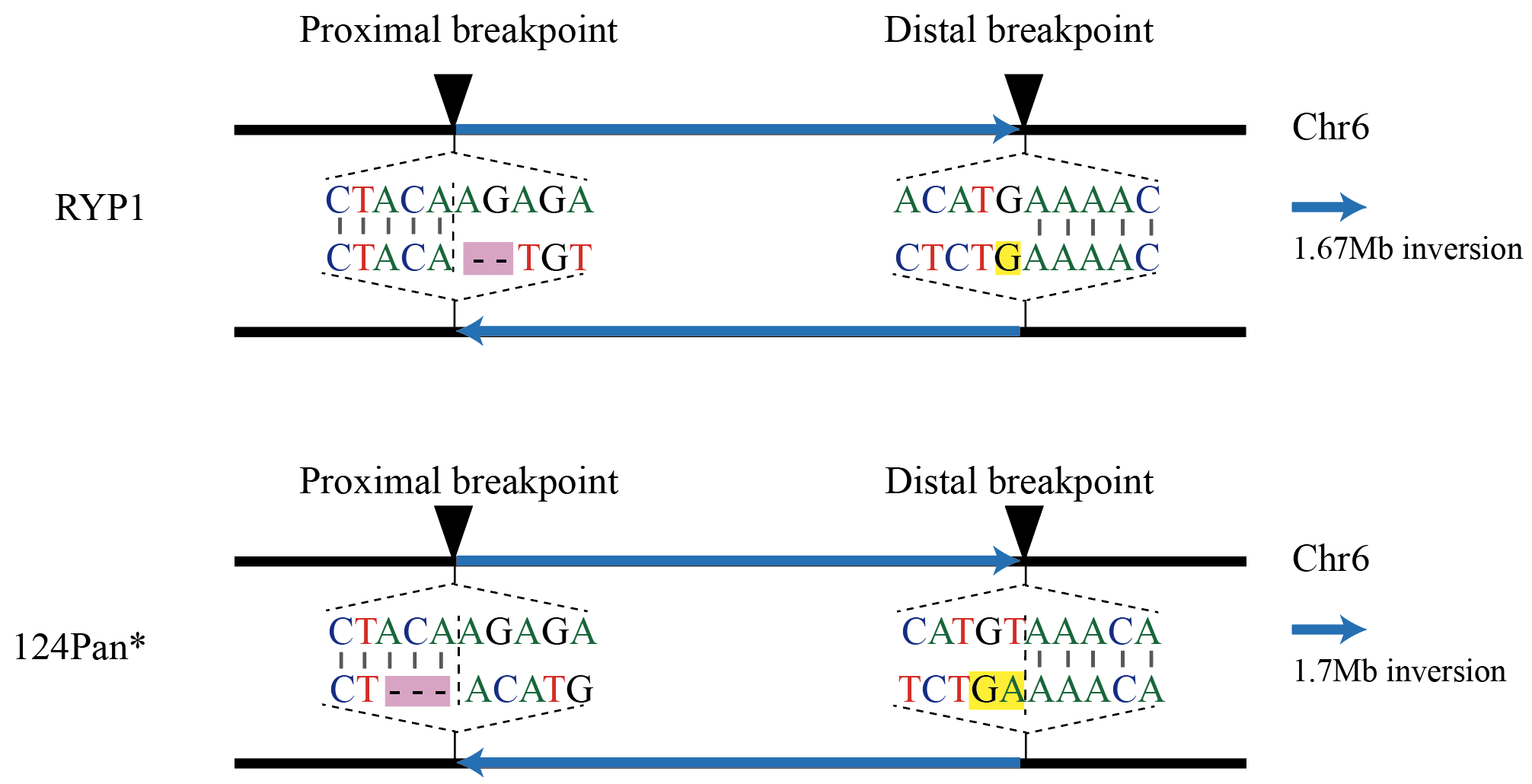


**Fig S18 Comparison of polymorphisms flanking the breakpoints of the heterozygous inversion between RYP1 and ‘124 Pan’ peach cultivars.** The shadow rectangle represents deletions (pink color) and insertions (yellow color) between the ancestral allele and the derived allele. * The polymorphism information here was taken from Zhou et al. [80].


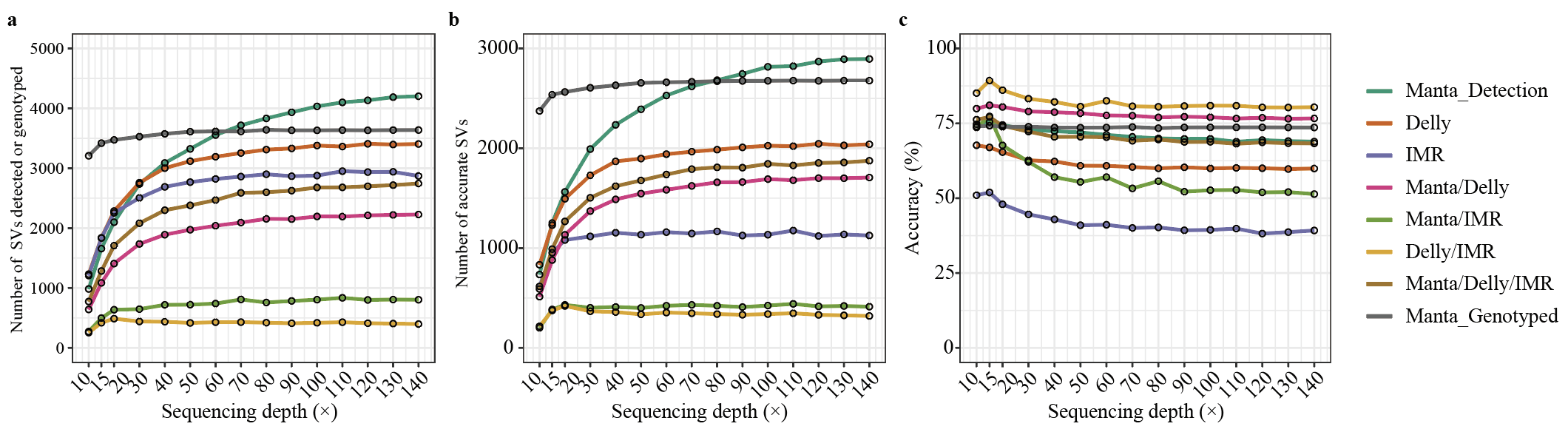


**Fig S19 Evaluation of SV caller programs using simulated short-read data.** Comparisons of the numbers and accuracy of SVs as *de novo* detected or genotyped based on different sequencing depths (×) of data randomly sampled from 140× Illumina short-read data of RYP1 using different SV caller programs. **a**, Number of SVs *de novo* detected using Manta (Manta_Detection), Delly, and IMR/DENOM (IMR), or combinations of these three programs (Manta/Delly, Manta/IMR, Delly/IMR, Manta/Delly/IMR), or genotyped based on reference SV set using Paragraph program (Manta_Genotyped). Note: the reference used to be genotyped was the *de novo* SV set called using Manta based on 140× short-read data. **b**, Number of accurate SVs when compared with the SVs set called based on high-depth (~600×) PacBio long reads of RYP1. Note: we considered an SV “accurate” if it overlapped (80% reciprocal overlap) with an SV called based on the PacBio long reads. **c**, Accuracy of different SV callers and combinations.
