# Supplementary material for: Genome structure variation analyses of peach reveal population dynamics and a 1.67 Mb causal inversion for fruit shape
Source: Genome Biol. 2021 Jan 5;22:13. doi: 10.1186/s13059-020-02239-1 (PMC7784018; doi:10.1186/s13059-020-02239-1)
Supplement: Supplementary file 2 — Additional file 2: Supplementary Tables S1–S7, S10–S12, S14. This file contains the supplementary tables referenced in the main text. Table S1. Summary of sequencing data. Table S2. Number and size of each chromosome (Chr) and unanchored contigs for the RYP1 genome. Table S3. Statistics for contigs/scaffolds that could not be anchored onto chromosomes in the RYP1/Lovell v2.0 genome assemblies. Table S4. The genome assembly and annotation completeness of the RYP1 and Lovell v2.0 genomes assessed by BUSCO. Table S5. Summary of statistics for transcriptional data from RNA sequencing (RNA-seq) analysis of different tissues for gene model prediction. Table S6. Annotation statistics of predicted protein-coding genes for the RYP1 genome. Table S7. Summary of repeat elements identified in the RYP1 genome and the Lovell v2.0 genome. Table S10. Summary statistics of orthogroups between the RYP1 and Lovell v2.0 genomes. Table S11. Summary of duplicated genes in the RYP1 and the Lovell v2.0 genomes. Table S12. Summary statistics of heterozygous variants (SNPs, InDels and SVs) in the RYP1 genome. Table S14. Number and length of different types of SVs in peach. [file 13059_2020_2239_MOESM2_ESM.docx]

**Table S1:** Summary of sequencing data.

| **Category** | **PacBio** | **Illumina** |
| --- | --- | --- |
| Library | 20 kb | 350 bp |
| Platform | Sequel II | Novaseq |
| Read number | 7,253,514 | 235,810,326 |
| Total length (Gb) | 140.98 | 35.37 |
| Depth* | 589.74× | 147.97× |
| N50 of read length (bp) | 30,451 | - |
| Mapping rate | - | 98.82% |

* The depth was calculated using the RYP1 genome assembly as reference genome

**Table S2:** Number and size of each chromosome (Chr) and unanchored contigs for the RYP1 genome.

|  | **Chr** | **Number** | **Size (bp)** |
| --- | --- | --- | --- |
| Pseudomolecules  Total | 1 | 7 | 49,444,689 |
|  | 2 | 9 | 32,391,467 |
|  | 3 | 14 | 28,898,652 |
|  | 4 | 9 | 27,074,866 |
|  | 5 | 6 | 18,727,589 |
|  | 6 | 6 | 31,983,997 |
|  | 7 | 5 | 22,860,706 |
|  | 8 | 6 | 23,625,283 |
|  |  | 62 | 235,007,249 |
| Unanchored | - | 25 | 4,045,591 |
| Total | - | 87 | 239,052,840 |

**Table S3:** Statistics for contigs/scaffolds that could not be anchored onto chromosomes in the RYP1/Lovell v2.0 genome assemblies.

|  | **RYP1** | **Lovell v2.0** |
| --- | --- | --- |
| Number of contigs/scaffolds | 25 | 183 |
| Total length of contigs/scaffolds (Mb) | 4.05 | 1.72 |
| Number of protein-coding genes | 439 | 62 |

**Table S4:** The genome assembly and annotation completeness of the RYP1 and Lovell v2.0 genomes assessed by BUSCO.

|  | **Genome** | | | | **Gene** | | | |
| --- | --- | --- | --- | --- | --- | --- | --- | --- |
|  | **RYP1** | | **Lovell v2.0** | | **RYP1** | | **Lovell v2.0** | |
|  | Number | Percent | Number | Percent | Number | Percent | Number | Percent |
| Complete BUSCOs | 1,402 | 97.4 | 1,405 | 97.6 | 1,352 | 93.9 | 1,351 | 93.8 |
| Complete and single-copy BUSCOs | 1,371 | 95.2 | 1,386 | 96.3 | 1,246 | 86.5 | 1,272 | 88.3 |
| Complete and duplicated BUSCOs | 31 | 2.2 | 19 | 1.3 | 106 | 7.4 | 79 | 5.5 |
| Fragmented BUSCOs | 11 | 0.8 | 11 | 0.8 | 56 | 3.9 | 58 | 4 |
| Missing BUSCOs | 27 | 1.8 | 24 | 1.6 | 32 | 2.2 | 31 | 2.2 |

**Table S5:** Summary of statistics for transcriptional data from RNA sequencing (RNA-seq) analysis of different tissues for gene model prediction.

| **Sample** | **Tissue** | **Stage (DAA)** | **Number of raw reads** | **Number of clean reads** | **Clean bases (Gb)** | **Q20** | **Q30** | **GC (%)** | **NCBI Accession** |
| --- | --- | --- | --- | --- | --- | --- | --- | --- | --- |
| RRB19538* | Flower | 7 | 51,168,678 | 50,414,018 | 7.56 | 97.68 | 93.53 | 45.70 | - |
| RRB19586* | Fruit | 20 | 44,052,154 | 43,467,242 | 6.52 | 96.89 | 91.76 | 45.34 | - |
| RRB19635* | Fruit | 50 | 45,004,572 | 44,549,240 | 6.68 | 96.8 | 91.74 | 45.68 | - |
| RRB19678* | Fruit | 86 | 44,426,518 | 43,110,020 | 6.47 | 96.71 | 91.39 | 45.95 | - |
| SAMEA3861653 | Root | - | - | - | - | - | - | - | SAMEA3861653 |
| SAMEA3861654 | Root | - | - | - | - | - | - | - | SAMEA3861654 |
| SAMEA3861655 | Root | - | - | - | - | - | - | - | SAMEA3861655 |
| SAMEA3861656 | Root | - | - | - | - | - | - | - | SAMEA3861656 |
| SAMEA3861657 | Root | - | - | - | - | - | - | - | SAMEA3861657 |
| SAMEA3861658 | Root | - | - | - | - | - | - | - | SAMEA3861658 |
| SAMEA3861659 | Leaves | - | - | - | - | - | - | - | SAMEA3861659 |
| SAMEA3861660 | Leaves | - | - | - | - | - | - | - | SAMEA3861660 |
| SAMEA3861661 | Leaves | - | - | - | - | - | - | - | SAMEA3861661 |
| SAMEA3861662 | Leaves | - | - | - | - | - | - | - | SAMEA3861662 |
| SAMEA3861663 | Leaves | - | - | - | - | - | - | - | SAMEA3861663 |
| SAMEA3861664 | Leaves | - | - | - | - | - | - | - | SAMEA3861664 |

DAA: Days after anthesis. * newly sequenced samples in this study.

**Table S6:** Annotation statistics of predicted protein-coding genes for the RYP1 genome.

| **Category** | **Number** | **Percentage** |
| --- | --- | --- |
| Protein-coding genes | 32,604 | 100.00% |
| NCBI nr | 29,149 | 89.40% |
| Swiss-Prot | 19,897 | 61.03% |
| InterPro | 22,173 | 68.01% |
| KEGG | 6,420 | 19.69% |
| GO | 16,235 | 49.79% |
| Total functionally annotated | 29,181 | 89.50% |
| Unannotated | 3423 | 10.50% |

**Table S7:** Summary of repeat elements identified in the RYP1 genome and the Lovell v2.0 genome.

| **Class** | **Subclass** | **RYP1** | | | **Lovell v2.0*** | | |
| --- | --- | --- | --- | --- | --- | --- | --- |
|  |  | **Number** | **Length (bp)** | **Percent** | **Number** | **Length (bp)** | **Percent** |
| Retrotransposons |  | 110,925 | 48,924,207 | 20.47% | 98,388 | 45,674,022 | 20.08% |
|  | LTR elements | 101,153 | 46,309,494 | 19.37% | 89,291 | 42,717,591 | 18.78% |
|  | Gypsy | 41,539 | 22,652,373 | 9.48% | 34,904 | 21,440,018 | 9.43% |
|  | Copia | 32,322 | 17,619,678 | 7.37% | 26,834 | 15,140,168 | 6.66% |
|  | Others | 27,292 | 6,037,443 | 2.53% | 27,553 | 6,137,405 | 2.70% |
|  | Non-LTR elements | 9,772 | 2,614,713 | 1.09% | 9,097 | 2,956,431 | 1.30% |
|  | SINEs | 2,995 | 386,929 | 0.16% | 2,404 | 217,423 | 0.10% |
|  | LINEs | 6,777 | 2,227,784 | 0.93% | 6,693 | 2,739,008 | 1.20% |
| DNA transposons |  | 74,927 | 31,619,326 | 13.23% | 68,128 | 30,523,402 | 13.42% |
|  | CMC-EnSpm | 13,519 | 11,173,585 | 4.67% | 11,944 | 12,276,988 | 5.40% |
|  | MULE-MuDR | 13,495 | 5,278,910 | 2.21% | 13,903 | 5,684,955 | 2.50% |
|  | PIF-Harbinger | 13,866 | 5,164,590 | 2.16% | 9,796 | 4,367,392 | 1.92% |
|  | hAT-Ac | 7,366 | 2,873,818 | 1.20% | 6,569 | 2,171,814 | 0.96% |
|  | Helitron | 6,606 | 2,519,283 | 1.05% | 2,700 | 974,989 | 0.43% |
|  | Others | 20,075 | 4,609,140 | 1.93% | 23,216 | 5,047,264 | 2.22% |
| Other repeats |  | 183,210 | 34,468,089 | 14.42% | 164,286 | 25,797,002 | 11.34% |
|  | Unkown | 94,986 | 29,576,528 | 12.37% | 77,634 | 21,852,482 | 9.61% |
|  | Satellites | 336 | 144,985 | 0.06% | 362 | 38,220 | 0.02% |
|  | Simple repeats | 72,242 | 2,848,778 | 1.19% | 70,961 | 2627,706 | 1.16% |
|  | Low complexity | 13,232 | 632,790 | 0.26% | 13,177 | 633,520 | 0.28% |
|  | Others | 2,414 | 1,265,008 | 0.53% | 2,152 | 645,074 | 0.28% |
| Total |  | 369,062 | 115,011,622 | 48.11% | 330,802 | 101,994,426 | 44.85% |

*Re-annotated repeat sequences using the same pipeline as used for the RYP1 genome.

**Table S10:** Summary statistics of orthogroups between the RYP1 and Lovell v2.0 genomes.

| **Category** | **RYP1** | **Lovell v2.0** |
| --- | --- | --- |
| Number of genes | 32,604 | 31,972 |
| Number of genes in orthogroups | 32,119 | 31,395 |
| Percentage of genes in orthogroups | 98.5 | 98.2 |
| Number of orthogroups containing two species | 31,347 | 30,815 |
| Percentage of orthogroups containing two species | 96.1 | 96.4 |
| Number of species-specific orthogroups | 103 | 87 |
| Number of genes in species-specific orthogroups | 772 | 580 |
| Percentage of genes in species-specific orthogroups | 2.4 | 1.8 |
| Number of expanded orthogroups | 1,590 | 992 |
| Number of genes in expanded orthogroups | 5,096 | 3,571 |
| Percentage of genes in expanded orthogroups | 15.6 | 11.2 |
| Number of unassigned genes | 485 | 577 |
| Percentage of unassigned genes | 1.5 | 1.8 |

**Table S11:** Summary of duplicated genes in the RYP1 and the Lovell v2.0 genomes.

| **Category** | **RYP1** | **Lovell v2.0** |
| --- | --- | --- |
| Singleton | 4,713 | 4,944 |
| Dispersed | 15,750 | 16,230 |
| Proximal | 3,017 | 2,977 |
| Tandem | 3,853 | 3,829 |
| Segmental | 5,271 | 3,992 |
| Total | 32,604 | 31,972 |

**Table S12:** Summary statistics of heterozygous variants (SNPs, InDels and SVs) in the RYP1 genome. Values outside parentheses indicate the number of SVs overlapping with genes; values inside parentheses indicate the number of genes overlapping with SVs.

| **Type** | **Number** | **Length (bp)** | **CDS overlap** |
| --- | --- | --- | --- |
| SNPs | 504,069 | 504,069 | - |
| InDels | 106,695 | 386,894 | - |
| SVs | 11,480 | 23,145,882 | 1,322(2,244) |
| Deletions | 5,182 | 10,426,320 | 854(1,195) |
| Duplications | 699 | 1,717,512 | 196(256) |
| Insertions | 5,578 | 7,547,551 | 262(239) |
| Inversions | 21 | 3,454,499 | 10(554) |

**Table S14:** Number and length of different types of SVs in peach.

| **Type** | **Number** | **Max length (bp)** | **Mean length (bp)** | **Median length (bp)** | **Min length (bp)** |
| --- | --- | --- | --- | --- | --- |
| Deletions | 15,138 | 75,809 | 1,710 | 198 | 31 |
| Insertions | 10,882 | 17,343 | 596 | 157 | 31 |
| Duplications | 1558 | 33,190 | 2,501 | 234.5 | 31 |
| Inversions | 156 | 1,674,224 | 14,246 | 1030 | 63 |
| Total | 27,734 | 1,674,224 | 1,388 | 173 | 31 |
