# Supplementary material for: Genome structure variation analyses of peach reveal population dynamics and a 1.67 Mb causal inversion for fruit shape
Source: Genome Biol. 2021 Jan 5;22:13. doi: 10.1186/s13059-020-02239-1 (PMC7784018; doi:10.1186/s13059-020-02239-1)
Supplement: Supplementary file 4 — Additional file 4. Review history. [file 13059_2020_2239_MOESM4_ESM.docx]

Review History

**First round of review**

**Reviewer 1**

**Are you able to assess all statistics in the manuscript, including the appropriateness of statistical tests used?** Yes, and I have assessed the statistics in my report.

**Comments to author:**

The manuscript 'Genomic Structure Variation Analyses of Peach Reveal Population Dynamics and a 1.67 Mb Causal Inversion for Fruit Shape' de novo assembled a high-quality genome for a flat fruit peach cultivar, and produced a comprehensive SV map for peach population. Furthermore,  the author uncovered a 1.67 Mbp inversion associated with fruit shape by SV-based GWAS. Candidate gene of PpPFP2 was overexpressed in tomato, and can be verified to control the flat fruit.
In general, this research provides a high quality peach genome, and the variations found in peach will contribute to genetic mechanism study for fruit shape, it deserve publication. However, in the current version of the manuscript, some issues still need to be declared and improved as follows,

1. The depth of sample re-sequencing varies greatly from 14x to 61x? Does different sequencing depth have an impact on SV results? The sequencing depth of more than 50 individuals is below 20x. Is this data sufficient for SV detection? Could you use data with different depth to analyze the adequacy and accuracy of SV detected, and provide the most appropriate depth for SV calling for the next generation sequencing in peach?

2. The author have obtained many very long SVs. As we know, the false positives of SVs obtained from the NGS data are relatively high. Researchers should be very careful about the accuracy of the SVs. Multiple software depending on different mechanism should be performed, such as read pair, splitting reads, assembly etc., which could significantly control the false positive rate. Software such as DELLY and IMR could be good choice. In addition, accurate rate of SNP calling and SV calling should be validate by experiment, and the loci of false positive should be explained.

3. Line 209, there are 149 + 37 peach accessions for the GWAS and heterozygous inversion validation, what are the genetic background for these peaches? How about the phylogenetic relationship and population structure? How about the distribution in the world? All these germplasm information will be interesting and helpful to understand the genetic variation derived.

4. Could you introduce more about the cultivar of 'RYP1' and 'Lovell'？The significance of different genetic background, heterozygosity, genome quality will contribute to understand the importance of this novel assembled genome. The two genome have different number of genes, and what are they? Can gene family or KEGG make it clear?

5. Regarding the variation of flat fruit shape of peach, author has emphasized that similar research results have been published on PBJ (doi:10.1111/PBI.13455), please cite it and discuss the result. In addition, comparisons should be carried out in detail with the previous study in peaches. Such as locations, types of mutation, candidate genes etc.

6. SV and SNP data sets can provide good resources for the scientific community, and the author need to provide your updated data in the form of attachments. Genome data and resequencing data with clear ID need to be uploaded to NCBI, and the accession number should be provided in the manuscript. In addition, I suggest that the author provide a friendly website to make a lot of useful data, including genome, SNP, and SV data easier for researchers to use.

7. Scripts and pipelines for the SNP/SV calling and related analyses should be provided for other researchers to reproduce the results.

Minor points,
Line 152:  Please add the reference for the Levell v2.0 genome.
Line 545-563:  It is confused that 189 accessions were used for SV merging, and only 186 accession were done for the mapping.
Line 843:  Ref. 42 is incomplete.

**Reviewer 2**

**Are you able to assess all statistics in the manuscript, including the appropriateness of statistical tests used?** Yes, and I have assessed the statistics in my report.

**Comments to author:**

It was a pleasure to review the work submitted by Guan and co-authors. They describe the assembly of a high-quality chromosome-scale of a peach accession, the identification of heterozygous structural variants (SV), population-scale SV patterns, and they provide compelling evidence that an inversion leads to the expression changes to PpOFP2, which contributes to fruit shape. The manuscript is overall well written and experiments are well described. By establishing a functional link between an SV and an agronomic trait, it's an important contribution to the field of crop genomics.

I have a series of suggestions that I think would improve the manuscript for publication.

First and foremost, all data should be made available. I haven't found mention of submission to public databases in the document I reviewed. The genome assembly, the sequencing reads, and possibly also vcf files should be made available before the work is accepted for publication. VCF files could be submitted to Zenodo or similar databases. The primers used for the qRT-PCR should be presented. I apologize if they were deposited and I missed it.

"Background"

Line 40 - Add citation to the first sentence
Line 46 - "protein-coding", missing "genes"?
Line 56 - Consider re-writing the sentence, long and not clear.
Line 60 - "as an abundant resource", I don't understand this, consider rewriting
Line 61 - "contributes" should be "contribute" since the subject (heterozygous SVs) is plural
Line 65 - start new paragraph after "impact desirable traits"
Lines 70-74 - Consider re-writing the sentence, long and not clear.
Lines 83-87 - Consider re-writing the sentence, long and not clear
Line 88 - "explain very well", can you use a more "scientific" verb?
Line 92 - It's the first time I see "ultra-high-depth", I recommend to refer to "high-depth" instead
Line 94 - remove "So", not necessary, I think
Line 97 - Consider rewriting, "facilitate us to" doesn't sound right.

"Results"

The first section on genome assembly & annotation could be significantly shortened by moving many of the details to the methods section.
I am curious why they didn't do polishing after CANU, but only after HERA. It could have improved the super-contigs.
Should the products of HERA be called scaffolds and not contigs?

Line 120 - It would be helpful to know the size of the unplaced sequences (i.e. sequences that couldn't be anchored and place in chromosomes) and how it compares to the Lovell genome.
Line 163 - I would be careful when discussing colinearity between the two genomes since the RYP1 chromosomes were built by anchoring on Lovell and therefore the two genomes are not independent of each other.
Lines 180-182 - "Given that tendemly..." I don't follow the logic here, consider re-writing.
Line 186 - consider rewriting, "hugely functionally impactful" doesn't sound right
Line 187-189 - I find the comment unnecessary in the context of the results.
Line 192 - Remove "thusly"
Line 206-208 - Rewrite, style/grammar issues
Line 215 - "reliable output"; I am not sure I would include such a comment about the output of Manta unless it's validated experimentally.
Line 259 - Is there a more "biochemical" annotation to the gene that Lr10-like or rust resistance gene? Lr if I am not mistaken refers to Leaf rust of cereals.
Lines 300-310. I am not convinced by the assumption that ALL SVs in cultivars are favorable for improvements. I recommend avoiding the use of "favorable vs. unfavorable" unless experimentally confirmed. There are plenty of examples of losses of important traits due to breeding, ranging from flavor to disease/stress tolerance.

**Authors Response**

**Point-by-point responses to the reviewers’ comments:**

**Reviewer #1:** The manuscript 'Genomic Structure Variation Analyses of Peach Reveal Population Dynamics and a 1.67 Mb Causal Inversion for Fruit Shape' de novo assembled a high-quality genome for a flat fruit peach cultivar, and produced a comprehensive SV map for peach population. Furthermore, the author uncovered a 1.67 Mbp inversion associated with fruit shape by SV-based GWAS. Candidate gene of PpPFP2 was overexpressed in tomato, and can be verified to control the flat fruit.
In general, this research provides a high-quality peach genome, and the variations found in peach will contribute to genetic mechanism study for fruit shape, it deserve publication. However, in the current version of the manuscript, some issues still need to be declared and improved as follows,

**1.** The depth of sample re-sequencing varies greatly from 14x to 61x? Does different sequencing depth have an impact on SV results? The sequencing depth of more than 50 individuals is below 20x. Is this data sufficient for SV detection? Could you use data with different depth to analyze the adequacy and accuracy of SV detected, and provide the most appropriate depth for SV calling for the next generation sequencing in peach?

**Response:**

First, we would like to thank the reviewer for the supportive comments and excellent guidance about how to improve our study. Regarding this comment specifically, the reviewer raises an important question. We chose Manta [1] as the SV caller in our analysis because of its high precision (low false discovery rate) and high recall, as demonstrated previously in a comprehensive evaluation study of ten highly cited SV callers [2].

To investigate the effect of sequencing depth on comprehensiveness and accuracy of *de novo* SV detection, we used the highest depth resequencing data (~150×; from the RYP1 cultivar) to sample different depths (10×, 15×, 20×, 30×, 40×, 50×, 60×, 70×, 80×, 90×, 100×, 110×, 120×, 130×, 140×) data. When using Manta and other two programs (Delly [3] and IMR/DENOM [4]) to detect SVs, the number of SVs detected was indeed affected greatly by the depth (**Figure 1a**), a trend that has also been observed in other study of evaluations for ten SV callers [2]. Next, we used the SVs detected using our high depth (~600×) PacBio long reads as the high-confidence set to approximately estimate the accuracy of SVs called for different depths of our Illumina data [2,5]. We considered an SV “accurate” if it overlapped (80% reciprocal overlap) with an SV called based on the PacBio long reads. The comparison results showed that the accuracy of SVs was not sensitive to the different depths (10×–140×) (**Figure 1c**). We therefore conclude that an appropriate depth for *de novo* SV calling for a single sample using Illumina PE150 data for peach is ~100×.

It also bears emphasis that our study included SV calling at the population scale (*i.e.*, including hundreds of samples) based on another strategy as performed by other recent studies [6-8]: genotyping of a reference SV set. First, we established reference SV set by merging the *de novo* detected SVs from data including the PacBio long reads and the Illumina NGS short reads from all samples in the population we examined. Second, based on the reference SV set and Illumina data, we used the graph-based genotyping program Paragraph [9] to identify the genotypes for all the samples. To validate the sensitivity and accuracy of this strategy, we used the SVs *de novo* detected using 140× Illumina data as the reference set, and genotyped the SV sites using the aforementioned differential depth datasets (10×–140×). Compared with numbers of the *de novo* detected SVs using Manta program, the number of genotyped SVs was not sensitive to the depth of the data (**Figure 1a**). The number of genotyped SVs based on 15× data was about three-fold higher than that of *de novo* detected SVs based on the same 15× data and almost equal to that based on > 60× data. In addition, the accuracy of genotyped SVs was also as high as that of the *de novo* method. Therefore, although the depths for our data ranged from 14×–61×, the comprehensiveness and accuracy of SVs is apparently quite high.

**Figure 1** Comparisons of the numbers and accuracy of SVs as *de novo* detected or genotyped based on different sequencing depths (×) of Illumina short-read data using different SV caller programs. **a**, Number of SVs *de novo* detected using Manta [2], Delly [3], and IMR/DENOM [4], or combinations of these three programs (or genotyped using Paragraph). Note: the reference was the *de novo* SV set called using Manta based on 140× short read data. **b**, Number of accurate SVs when compared with the SVs set called based on high-depth (~600×) PacBio long reads. **c**, Accuracy of different SV callers and combinations.

**References:**

1. Chen X, Schulz-Trieglaff O, Shaw R, Barnes B, Schlesinger F, Kallberg M, Cox AJ, Kruglyak S, Saunders CT. Manta: rapid detection of structural variants and indels for germline and cancer sequencing applications. Bioinformatics. 2016;32(8):1220–1222.
2. Cameron DL, Di Stefano L, Papenfuss AT. Comprehensive evaluation and characterization of short read general-purpose structural variant calling software. Nature Commun. 2019;10(1):3240.
3. Rausch T, Zichner T, Schlattl A, Stütz AM, Benes V, Korbel JO. DELLY: structural variant discovery by integrated paired-end and split-read analysis. Bioinformatics. 2012;28(18):i333–i339.
4. Gan X, Stegle O, Behr J, Steffen JG, Drewe P, Hildebrand KL, Lyngsoe R, Schultheiss SJ, Osborne EJ, Sreedharan VT, et al. Multiple reference genomes and transcriptomes for *Arabidopsis thaliana*. Nature. 2011;477(7365):419–423.
5. Sedlazeck FJ, Rescheneder P, Smolka M, Fang H, Nattestad M, von Haeseler A, Schatz MC. Accurate detection of complex structural variations using single-molecule sequencing. Nat Methods. 2018;15(6):461–468.
6. Chander V, Gibbs RA, Sedlazeck FJ. Evaluation of computational genotyping of structural variation for clinical diagnoses [Internet]. GigaScience. 2019; Available from: <https://doi.org/10.1093/gigascience/giz110>.
7. Yang N, Liu J, Gao Q, Gui S, Chen L, Yang L, Huang J, Deng T, Luo J, He L, et al. Genome assembly of a tropical maize inbred line provides insights into structural variation and crop improvement. Nat Genet. 2019;51(6):1052–1059.
8. Liu Y, Du H, Li P, Shen Y, Peng H, Liu S, Zhou G, Zhang H, Liu Z, Shi M, et al. Pan-Genome of wild and cultivated soybeans. Cell. 2020;182(1):162–176.e13.
9. Chen S, Krusche P, Dolzhenko E, Sherman RM, Petrovski R, Schlesinger F, Kirsche M, Bentley DR, Schatz MC, Sedlazeck FJ, et al. Paragraph: a graph-based structural variant genotyper for short-read sequence data. Genome Biol. 2019;20(1):291.

**2.** The author have obtained many very long SVs. As we know, the false positives of SVs obtained from the NGS data are relatively high. Researchers should be very careful about the accuracy of the SVs. Multiple software depending on different mechanism should be performed, such as read pair, splitting reads, assembly etc., which could significantly control the false positive rate. Software such as DELLY and IMR could be good choice. In addition, accurate rate of SNP calling and SV calling should be validate by experiment, and the loci of false positive should be explained.

**Response:**

Thanks for this helpful guidance. Before describing the results from our new Delly and IMR/DENOM analyses, we would like to highlight that the *de novo* SV caller Manta does combine different forms of input evidence (including read-pair, split-read, and also assembly) to identify high-quality SVs [1]. We have now performed analyses with the two programs (Delly [2] and IMR/DENOM [3]) suggested by reviewer with the differential depth Illumina datasets (using default parameters). The SVs called by Manta and Delly were filtered by retaining the SVs with a “PASS” tag. Comparisons of the program outputs showed that Manta generally outperformed Delly and IMR/DENOM for sensitivity (especially when the depth was higher than 50×) and accuracy (**Figure 1a,b,c**).

Next, we assessed the overlapping SVs in any two programs (or at least two programs when we merged SVs from three programs). Although the accuracy for the Delly/IMR combination and the Manta/Delly combination was slightly higher than that of Manta alone, the number of total SVs and accurate SVs called by Manta alone was much higher at all depths [1]. Notably, the combinations of three software performed worst. The advantage of Manta on sensitivity and accuracy may be attributed to its incorporation of different forms of input evidence and its excellent performance for detecting insertions [1].

To evaluate the accurate rate of SNP called based on resequencing data, we compared locations and genotypes of those SNP sites with those from our customized peach SNP genotyping array (a tool that was originally designed based on high-quality SNP sites characterized from extensive re-sequencing data). A total of 74,692 common sites for 126 accessions (Additional file 2: Table S22 of the revised manuscript) were overlapped. The validation rate for 126 accessions was 98.14–99.71% with a high total validation rate 99.18%, showing a low false positive rate (0.82%) of SNPs in the present study, which was comparable with the validation rates reported from other studies of sheep and maize resequencing efforts [4-5]. For SV validation, a total of 200 deletions and 100 insertions of 6 accessions from northwest, southwest, south, north China, Americas and Europe were selected to perform PCR-based validation. We estimated that the false discovery rate of total SVs, deletions and insertions was 5.77%, 5.69%, and 5.94% (Additional file 2: Table S15 of the revised manuscript), which was comparable with other studies [5-6].

**References**:

1. Chen X, Schulz-Trieglaff O, Shaw R, Barnes B, Schlesinger F, Kallberg M, Cox AJ, Kruglyak S, Saunders CT. Manta: rapid detection of structural variants and indels for germline and cancer sequencing applications. Bioinformatics. 2016;32(8):1220–1222.
2. Rausch T, Zichner T, Schlattl A, Stütz AM, Benes V, Korbel JO. DELLY: structural variant discovery by integrated paired-end and split-read analysis. Bioinformatics. 2012;28(18):i333–i339.
3. Gan X, Stegle O, Behr J, Steffen JG, Drewe P, Hildebrand KL, Lyngsoe R, Schultheiss SJ, Osborne EJ, Sreedharan VT, et al. Multiple reference genomes and transcriptomes for *Arabidopsis thaliana*. Nature. 2011;477(7365):419–423.
4. Li X, Yang J, Shen M, Xie X, Liu G, Xu Y, Lv F, Yang H, Yang Y, Liu C, et al. Whole-genome resequencing of wild and domestic sheep identifies genes associated with morphological and agronomic traits. Nat Commun. 2020;11(1):2815.
5. Yang N, Liu J, Gao Q, Gui S, Chen L, Yang L, Huang J, Deng T, Luo J, He L, et al. Genome assembly of a tropical maize inbred line provides insights into structural variation and crop improvement. Nat Genet. 2019;51(6):1052–1059.
6. Zhang Z, Mao L, Chen H, Bu F, Li G, Sun J, Li S, Sun H, Jiao C, Blakely R, et al. Genome-wide mapping of structural variations reveals a copy number variant that determines reproductive morphology in cucumber. Plant Cell. 2015;27(6):1595–1604.

**3.** Line 209, there are 149 + 37 peach accessions for the GWAS and heterozygous inversion validation, what are the genetic background for these peaches? How about the phylogenetic relationship and population structure? How about the distribution in the world? All these germplasm information will be interesting and helpful to understand the genetic variation derived.

**Response**:

We thank the reviewer for inviting us to elaborate on this essential information about our germplasm diversity panel. Accordingly, we have added new content to the Lines 217-223 of the revised manuscript to detail each of the information types mentioned in this review comment. We provide this information for all accessions in Additional file 1: Figure S6 and Additional file 2: Table S13 of the revised manuscript, and have now included phylogenetic and model-based clustering analysis in a revised manuscript. Specifically, the 37 *P. kansuensis* accessions, as the wild relatives of peach, were selected as the outgroup (OG). We chose 149 *P. persica* accessions from main producing areas or countries, including north, northwest, south, southwest China, Japan, the Americas and Europe [1]. Based on 60,405 SNP sites at fourfold degenerate sites (Missing rate < 50%, Minor allele frequency > 0.05), the phylogenetic tree was built using FastTree v2.1.10 [2] and population structure was inferred using ADMIXTURE v1.3.0 [3] for each K value from 2 to 4 with 1,000 bootstrap replicates. The results of these new analyses support the division of the panel into an outgroup, landraces, oriental modern cultivars, and occidental modern cultivars (**Figure 2**).

Figure 2. Phylogenetic tree and model-based clustering analysis of the population (149 *P. persica* accessions and 37 wild relatives, *P. kansuensis*, as the outgroup) constructed using 60,405 SNPs at fourfold degenerate sites (Missing rate < 50%, Minor allele frequency > 0.05).

**References**:

1. Guan L, Cao K, Li Y, Guo J, Xu Q, Wang L. Detection and application of genome-wide variations in peach for association and genetic relationship analysis. BMC Genet. 2019;20(1):101.
2. Price MN, Dehal PS, Arkin AP. FastTree: computing large minimum evolution trees with profiles instead of a distance matrix. Mol Biol Evol. 2009;26(7):1641–1650.
3. Alexander DH, Novembre J, Lange K. Fast model-based estimation of ancestry in unrelated individuals. Genome Res. 2009;19(9):1655–1664.

**4.** Could you introduce more about the cultivar of 'RYP1' and 'Lovell'？The significance of different genetic background, heterozygosity, genome quality will contribute to understand the importance of this novel assembled genome. The two genomes have different number of genes, and what are they? Can gene family or KEGG make it clear?

**Response:**

Thanks for the reviewer’s comment! We have taken this comment as an invitation to expand the context information for these cultivars in Lines 159-162 of our revised text. The sequenced Rui You Pan 1 (RYP1) is a flat, low acid, white flesh, nectarine cultivar. RYP1 has many phenotypes that are distinct from the main genomic model cultivar Lovell (the cultivar selected for the initial Peach genome sequencing project that was reported in 2013). Lovell is widely used as rootstock globally [1], and also has many traits attractive to North American buyers, including its round, acid, yellow flesh color, and glabrous skin (Additional file 2: Table S8 of the revised manuscript).

We have performed gene clustering between the RYP1 and Lovell genomes based on protein sequences using OrthoFinder v2.3.9 [2]. As described at Lines 171-184 in the updated manuscript, a total of 103 and 87 specific orthogroups containing 772 and 580 genes were identified as specific to the RYP1 or the Lovell genomes, respectively. In addition, we also found that 992 and 1,590 expansion orthogroups containing 3,571 and 5,096 genes displayed higher copy numbers of genes in the Lovell v2.0 or the RYP1 genomes, respectively. KEGG (Kyoto Encyclopedia of Genes and Genomes) enrichment analysis was further performed for the specific and expansion orthogroups. The results showed RYP1-specific enrichment for a “fructose and mannose metabolism” pathway (ko00051) **(Figure 3a,b**). Detailed examination of the genes in this pathway displayed that two genes related to fructose metabolism, sorbitol dehydrogenase (*SDH*) and pyrophosphate fructose-6-phosphate 1-phosphotransferase subunit beta (*PFPβ*), had higher copy numbers in the RYP1 genome (3 and 4 copies) than in the Lovell v2.0 genome (2 and 3). The SDH gene product uses sorbitol as a substrate and produces fructose [3], and the activity of SDH has been reported as responsible for regulation of the fructose concertation in peach fruit [4]. And PFPβ is known to be one of the two subunits of the PFP enzyme complex that catalyzes the glycolytic pathway reaction from fructose-6-phosphate (F6P) to fructose-1,6-bisphosphate (FBP); this complex also supports gluconeogenesis [5-6]. The activity of PFP has been associated with the sugar content in in plants including sugarcane [7], sweet pepper [8], and strawberry [9].

**Figure 3** Kyoto Encyclopedia of Genes and Genomes (KEGG) pathway analysis of Lovell (**a**) and RYP1 (**b**) specific and expansion genes, and the phylogenetic tree of expansion genes related to the fructose and mannose metabolism (**c**). X-axis represents the ratio of the enriched gene number among the total analyzed genes. The size of the round nodes is in proportion to the number of genes significantly enriched in each pathway. Color of round node represents the P value of significant KEGG pathway. Sorbitol dehydrogenase (*SDH*). Pyrophosphate fructose-6-phosphate 1-phosphotransferase subunit beta (*PFPβ*).

**References**:

1. Wheeler W, Wytsalucy R, Black B, Cardon G, Bugbee B. Drought tolerance of Navajo and Lovell peach trees: Precision water stress using automated weighing lysimeters, HortScience. 2019;54(5):799–803.
2. Emms DM, Kelly S. OrthoFinder: phylogenetic orthology inference for comparative genomics. Genome Biol. 2019;20(1):238.
3. Aguayo MF, Ampuero D, Mandujano P, Parada R, Muñoz R, Gallart M, Altabella T, Cabrera R, Stange C, Handford M. Sorbitol dehydrogenase is a cytosolic protein required for sorbitol metabolism in *Arabidopsis thaliana*. Plant Sci. 2013;205-206:63–75.
4. Kanayama Y, Kogawa M, Yamaguchi M, Kanahama K. Fructose content and the activity of fructose-related enzymes in the fruit of eating-quality peach cultivars and native-type peach cultivars. J Jpn Soc Hortic Sci. 2005;74(6):431–436.
5. Plaxton WC. The organization and regulation of plant glycolysis. Annu Rev Plant Physiol Plant Mol Biol. 1996;47:185–214.
6. Nielsen TH, Rung JH, Villadsen D. Fructose-2,6-bisphosphate: a traffic signal in plant metabolism. Trends Plant Sci. 2004;9(11):556–563.
7. Whittaker A, Botha FC. Pyrophosphate: D-fructose-6-phosphate 1-phosphotransferase activity patterns in relation to sucrose storage across sugarcane varieties. Physiol Plant. 1999;107:379–386.
8. Nielsen TH, Skjærbæk HC, Karlsen P. Carbohydrate metabolism during fruit development in sweet pepper (*Capsicum annuum*) plants. Physiol. Plant. 1991;82(2):311–319.
9. Basson CE, Groenewald JH, Kossmann J, Cronje C, Bauer R. Sugar and acid‐related quality attributes and enzyme activities in strawberry fruits: invertase is the main sucrose hydrolysing enzyme. Food Chem. 2010;121(4):1156–1162.

**5.** Regarding the variation of flat fruit shape of peach, author has emphasized that similar research results have been published on PBJ (doi:10.1111/PBI.13455), please cite it and discuss the result. In addition, comparisons should be carried out in detail with the previous study in peaches. Such as locations, types of mutation, candidate genes etc.

**Response:**

Thanks; we have now cited the relevant study and discuss it in detail in Lines of 459-470 in our revised manuscript. The Zhou et al*.* [1] study focused on mapping of causal genes for flat fruit shape and used the Lovell v2.0 genome as the reference. They also reported that a 1.7 Mb inversion on chromosome Pp06 co-segregated with flat shape, and showed that overexpression of *PpOFP1* (upstream of the inversion) is responsible for the flat shape, with experimental data support from Arabidopsis [1]. We are pleased that this similar work could support one of our findings about the flat-shape-control function of *PpOFP2* based on our SV map.

Compared with their study, and based on our assembly of the flat peach genome RYP1, we reported a shorter (more accurate) inversion (~1.67 Mb) with no gaps. And based on the PCR and Sanger sequencing data, we found that there is a two-nucleotide deletion flanking the proximal breakpoint and a one-nucleotide insertion flanking the distal breakpoint for the RYP1 cultivar; this differs from their finding for the ‘124 Pan’ flat cultivar (a three-nucleotide deletion flanking the proximal breakpoint and a two-nucleotide insertion flanking the distal breakpoint). In addition, we also mapped the same causal gene belong to OVATE family; however, we named it as *PpOFP2* because it is an ortholog of *AtOFP2*. Notably, we measured the expression of the *PpOFP2* gene in a much larger population including 37 flat and 99 round peach cultivars. Moreover, and based on the eGWAS method, we further identified tight associations between up-regulated expression of *PpOFP2* with the heterozygous inversion. Finally, our validation of the negative regulatory function of *PpOFP2* was with the model fruit species tomato rather than Arabidopsis.

**References**:

1. Zhou H, Ma R, Gao L, Zhang J, Zhang A, Zhang X, Ren F, Zhang W, Liao L, Yang Q, et al. A 1.7-Mb chromosomal inversion downstream of a *PpOFP1* gene is responsible for flat fruit shape in peach. Plant Biotechnol J. 2020. doi: 10.1111/pbi.13455. Epub ahead of print. PMID: 32722872.

**6.** SV and SNP data sets can provide good resources for the scientific community, and the author need to provide your updated data in the form of attachments. Genome data and resequencing data with clear ID need to be uploaded to NCBI, and the accession number should be provided in the manuscript. In addition, I suggest that the author provide a friendly website to make a lot of useful data, including genome, SNP, and SV data easier for researchers to use.

**Response**:

Yes, we are excited to release our data for the peach and tree fruit genomics research communities. The raw resequencing data and transcriptome data have now been deposited in Sequence Read Archive (https://www.ncbi.nlm.nih.gov/sra/) of National Center for Biotechnology Information (NCBI) under BioProjects PRJNA663114 and PRJNA664002, respectively. This whole genome shotgun project has been deposited at GenBank under the accession JACYOW000000000. The raw PacBio data was available in the NCBI Sequence Read Archive under BioProject PRJNA663129. The SNP and SV vcf files can be freely and openly accessed on figshare (https://doi.org/10.6084/m9.figshare.12937340.v1).

7. Scripts and pipelines for the SNP/SV calling and related analyses should be provided for other researchers to reproduce the results.

**Response:** Yes, we are very pleased to provide our scripts and analysis pipelines for other researchers. We have included the pipeline used for SNP/SV calling, for our phylogenetic analysis, for GWAS, and additional analysis in a TXT file with comments about the parameters, and the scripts can be freely and openly accessed on figshare (https://doi.org/10.6084/m9.figshare.12937340.v1).

Minor points,
Line 152: Please add the reference for the Lovell v2.0 genome.

**Response:** Thanks for this comment! We have added the reference in Line 148 of the updated manuscript.

Line 545-563: It is confused that 189 accessions were used for SV merging, and only 186 accession were done for the mapping.

**Response:** Thanks for catching this, which was an error. We have now updated this to the correct number (186) in the revised manuscript.

Line 843: Ref. 42 is incomplete.
**Response:** Thanks; we now completed this reference (see Lines 920-921 of revised manuscript).

**Reviewer #2:** It was a pleasure to review the work submitted by Guan and co-authors. They describe the assembly of a high-quality chromosome-scale of a peach accession, the identification of heterozygous structural variants (SV), population-scale SV patterns, and they provide compelling evidence that an inversion leads to the expression changes to PpOFP2, which contributes to fruit shape. The manuscript is overall well written and experiments are well described. By establishing a functional link between an SV and an agronomic trait, it's an important contribution to the field of crop genomics.

I have a series of suggestions that I think would improve the manuscript for publication.

First and foremost, all data should be made available. I haven't found mention of submission to public databases in the document I reviewed. The genome assembly, the sequencing reads, and possibly also vcf files should be made available before the work is accepted for publication. VCF files could be submitted to Zenodo or similar databases. The primers used for the qRT-PCR should be presented. I apologize if they were deposited and I missed it.

**Response:**

First, thanks the reviewer for the supportive comments and the very helpful guidance about how to get our study ready for publication. Regarding this comment specifically, we have now carefully ensured that all of our data, material information, and computational tools are readily accessible via public databases and resources. The raw resequencing data and transcriptome data have now been deposited in Sequence Read Archive (https://www.ncbi.nlm.nih.gov/sra/) of National Center for Biotechnology Information (NCBI) under BioProjects PRJNA663114 and PRJNA664002, respectively. This whole genome shotgun project has been deposited at GenBank under the accession JACYOW000000000. The raw PacBio data was available in the NCBI Sequence Read Archive under BioProject PRJNA663129. The SNP and SV vcf files can be freely and openly accessed on figshare (https://doi.org/10.6084/m9.figshare.12937340.v1). The primer sequences are now included in the revised manuscript as Additional file 2: Table S23 of the revised manuscript.

"Background"

Line 40 - Add citation to the first sentence

**Response**: Thanks for this comment; we have added references in Line 40 of the updated manuscript, highlighted by yellow color.

Line 46 - "protein-coding", missing "genes"?

**Response**: Thanks for this comment; we have rewritten this sentence in Line 46 of the updated manuscript, highlighted by yellow color.

Line 56 - Consider re-writing the sentence, long and not clear.

**Response:** Thanks for this comment; we have rewritten the reference in Lines 51-58 of the updated manuscript, highlighted by yellow color.

Line 60 - "as an abundant resource", I don't understand this, consider rewriting

**Response**: Thanks for this comment! We have reworded this phrase in Lines 59-60 of the updated manuscript, highlighted by yellow color.

Line 61 - "contributes" should be "contribute" since the subject (heterozygous SVs) is plural

**Response**: Thanks for this comment; we have revised this word in Line 60 of the updated manuscript, highlighted by yellow color.

Line 65 - start new paragraph after "impact desirable traits"

**Response**: Thanks for this comment; we have started a new paragraph at Line 65 of the updated manuscript, highlighted by yellow color.

Lines 70-74 - Consider re-writing the sentence, long and not clear.

**Response**: Thanks for this comment; we have revised this sentence in Lines 70-71 of the updated manuscript, highlighted by yellow color.

Lines 83-87 - Consider re-writing the sentence, long and not clear

**Response**: Thanks for this comment; we have revised this sentence in Lines 79-82 of the updated manuscript, highlighted by yellow color.

Line 88 - "explain very well", can you use a more "scientific" verb?

**Response**: Thanks for this comment; we have revised this sentence in Lines 82-84 of the updated manuscript, highlighted by yellow color.

Line 92 - It's the first time I see "ultra-high-depth", I recommend to refer to "high-depth" instead

**Response**: Thanks for this comment! We have changed the word to “high-depth” here and other places.

Line 94 - remove "So", not necessary, I think

**Response**: Thanks for this comment! We have removed this word.

Line 97 - Consider rewriting, "facilitate us to" doesn't sound right.

**Response**: Thanks for this comment! We have modified this word in Lines 92-94 of the updated manuscript, highlighted by yellow color.

"Results"

The first section on genome assembly & annotation could be significantly shortened by moving many of the details to the methods section.

**Response**: Thanks for this comment! We considered deleting this, but have retained it for clarity of message. Given the enormous number of bioinformatics tools available for functional genomics work, we feel it is informative for casual readers to briefly present the basis of these analyses prior to introducing data.

I am curious why they didn't do polishing after CANU, but only after HERA. It could have improved the super-contigs.
Should the products of HERA be called scaffolds and not contigs?

**Response:**

Thanks for raising this issue. The strategy not to polish the contigs assembled by CANU program [1] was because the base accuracy of the consensus sequences corrected and assembled by CANU are typically well above 99% for PacBio datasets [1]. This high accuracy of assembly is enough for the HERA algorithm to further enhance the continuity of these contigs through assembly using corrected reads from CANU [2]. In addition, to our understanding, the purpose of doing polishing by next generation sequencing reads is primarily to correct for the small amount of single bases differences and small indels that may be remaining in the assembled genome. Therefore, we polished the final genome sequence after extended assembly of the HERA algorithm [2].

HERA was used to connect and fill gaps between the CANU-assembled contigs, specifically by traversing the overlap graph with corrected reads. As such, at least according to previous studies (see for example [3-4]) and our understanding, it is suitable to refer to products from HERA as “contigs”, and these contigs have no gaps.

**References**:

1. Koren S, Walenz BP, Berlin K, Miller JR, Bergman NH, Phillippy AM. Canu: scalable and accurate long-read assembly via adaptive k-mer weighting and repeat separation. Genome Res. 2017;27(5):722–736.
2. Du H, Liang C. Assembly of chromosome-scale contigs by efficiently resolving repetitive sequences with long reads. Nat Commun. 2019;10(1):5360.
3. Shen Y, Du H, Liu Y, Ni L, Wang Z, Liang C, Tian Z. Update soybean Zhonghuang 13 genome to a golden reference. Sci China Life Sci. 2019;62(9):1257–1260.
4. Shen C, Du H, Chen Z, Lu H, Zhu F, Chen H, Meng X, Liu Q, Liu P, Zheng L, et al. The chromosome-level genome sequence of the autotetraploid Alfalfa and resequencing of core germplasms provide genomic resources for Alfalfa research. Mol Plant. 2020;13(9):1250–1261.

Line 120 - It would be helpful to know the size of the unplaced sequences (i.e. sequences that couldn't be anchored and place in chromosomes) and how it compares to the Lovell genome.

**Response:**

We thank the reviewer for inviting us to elaborate on this useful information. The number of unplaced contigs in our RYP1 assembly is 25, comprising a total of 4.05 Mb. The number of unplaced scaffolds in Lovell v2.0 genome is 183, representing a total of 1.72 Mb. Because the total size and contiguity of the RYP1 genome is much than the Lovell v2.0 genome assembly, anchoring using Lovell v2.0 genome as a reference resulted in a situation where longer contigs are output as unplaced. We have provided this information in Additional file 2: Table S3 of the revised manuscript and note this in Lines 111-114 of the updated main text.

Line 163 - I would be careful when discussing collinearity between the two genomes since the RYP1 chromosomes were built by anchoring on Lovell and therefore the two genomes are not independent of each other.

**Response:**

Thanks for this guidance. There is an important distinction here: following the method for assessing collinearity from a previous study that included long-read data [1], we need to stress that although we anchored the contigs into chromosomes based on the Lovell v2.0 reference sequence, the assembly of the contig sequences themselves was independent of the reference sequence. These contigs were in general large (N50 11.5Mb, with each chromosome comprising only 6-14 contigs), and these had high collinearity (93.09%) with a genetic map. It is thus unlikely that we misplaced the contigs.

**References:**

1. Jiao WB, Schneeberger K. Chromosome-level assemblies of multiple *Arabidopsis* genomes reveal hotspots of rearrangements with altered evolutionary dynamics. Nat Commun. 2020;11(1):989.

Lines 180-182 - "Given that tendemly..." I don't follow the logic here, consider re-writing.

**Response:** Thanks for this comment! We have modified this sentence in Lines 190-191 of the updated manuscript, highlighted by yellow color.

Line 186 - consider rewriting, "hugely functionally impactful" doesn't sound right

**Response:** Thanks for this comment! We have modified this sentence in Line 194 of the updated manuscript, highlighted by yellow color.

Line 187-189 - I find the comment unnecessary in the context of the results.

**Response:** Thanks for this comment! We have deleted this comment in the updated manuscript.

Line 192 - Remove "thusly"

**Response:** Thanks for this comment! We have removed this word, which can be seen in Line 198 of updated manuscript, highlighted by yellow color.

Line 206-208 - Rewrite, style/grammar issues

**Response:** Thanks for this comment! We have modified this sentence in Lines 212-213 of the updated manuscript, highlighted by yellow color.

Line 215 - "reliable output"; I am not sure I would include such a comment about the output of Manta unless it's validated experimentally.

**Response:** Thanks for this comment; we have altered the text as guided. Please note that we have also now performed experimental validations for SVs called from Manta. A total of 200 deletions and 100 insertions of 6 accessions from northwest, southwest, south, north of China, Americas and Europe were selected to perform PCR-based validation. Based on the PCR data, we estimated that the false discovery rate of total SVs, deletions and insertions was 5.77%, 5.69% and 5.94% (Additional file 2: Table S15 of the revised manuscript), which was comparable with other studies in maize and cucumber [1-2].

**References**:

1. Yang N, Liu J, Gao Q, Gui S, Chen L, Yang L, Huang J, Deng T, Luo J, He L, et al. Genome assembly of a tropical maize inbred line provides insights into structural variation and crop improvement. Nat Genet. 2019;51(6):1052–1059.
2. Zhang Z, Mao L, Chen H, Bu F, Li G, Sun J, Li S, Sun H, Jiao C, Blakely R, et al. Genome-wide mapping of structural variations reveals a copy number variant that determines reproductive morphology in cucumber. Plant Cell. 2015;27(6):1595–1604.

Line 259 - Is there a more "biochemical" annotation to the gene that Lr10-like or rust resistance gene? Lr if I am not mistaken refers to Leaf rust of cereals.

**Response:**

Thanks for this helpful comment! Yes, while these genes were originally identified as orthologs of the wheat leaf rust resistance gene *LRK10*, we now know that this gene encodes a receptor-like protein kinase [1-2]. Accordingly, we have updated the text in Lines 272-273 to reflect this more informative “biochemical” functional annotation.

**References**:

1. Feuillet C, Schachermayr G, Keller B. Molecular cloning of a new receptor-like kinase gene encoded at the *Lr10* disease resistance locus of wheat. Plant J. 1997;11(1):45–52.
2. Feuillet C, Reuzeau C, Kjellbom P, Keller B. Molecular characterization of a new type of receptor-like kinase (wlrk) gene family in wheat. Plant Mol Biol. 1998;37(6):943–953.

Lines 300-310. I am not convinced by the assumption that ALL SVs in cultivars are favorable for improvements. I recommend avoiding the use of "favorable vs. unfavorable" unless experimentally confirmed. There are plenty of examples of losses of important traits due to breeding, ranging from flavor to disease/stress tolerance.

**Response:** As guided by this comment we have toned down claims about “all SVs” in the revise manuscript, and now employ the definitions for "favorable vs. unfavorable" SVs from a major study of the model fruit species tomato [1].

We would like to again thank the reviewer the careful analysis of our study, the supportive comments, and the excellent guidance about how to improve our study and the manuscript describing it. Many thanks.

**References**:
1.	Gao L, Gonda I, Sun H, Ma Q, Bao K, Tieman DM, Burzynski-Chang EA, Fish TL, Stromberg KA, Sacks GL, et al. The tomato pan-genome uncovers new genes and a rare allele regulating fruit flavor. Nat Genet. 2019;51(6):1044–1051.

**Second round of review**

**Reviewer 1**

Manuscript of "Genomic Structure Variation Analyses of Peach Reveal Population Dynamics and a 1.67 Mb Causal Inversion for Fruit Shape" (GBIO-D-20-01097R1) addressed most of my concerns. However, there are still two issues that should arouse the author's attention before final accept.：
First, Figure 1 in response 1 and related contents on adequacy and accuracy need to be included in the article, which can guide the later researchers.
Second, the script/pipelines provided by the author is too rough to reproduce the results. Please complete related details and refer to some excellent works, such as <https://doi.org/10.1038/s41588-020-0699-x>.

**Authors Response**

**Point-by-point responses to the reviewers’ comments:**

Reviewer #1: Manuscript of “Genomic Structure Variation Analyses of Peach Reveal Population Dynamics and a 1.67 Mb Causal Inversion for Fruit Shape” (GBIO-D-20-01097R1) addressed most of my concerns. However, there are still two issues that should arouse the author's attention before final accept.：
First, Figure 1 in response 1 and related contents on adequacy and accuracy need to be included in the article, which can guide the later researchers. 
Second, the script/pipelines provided by the author is too rough to reproduce the results. Please complete related details and refer to some excellent works, such as <https://doi.org/10.1038/s41588-020-0699-x>.

**Response:**

Thanks for this helpful guidance about how to improve our work. The figure 1 in the response letter has been included in our newly revised manuscript (Additional file 1: Fig. S19), and the relevent contents have been complemented in the Methods section under the subsection of ‘SNP, small InDel, and SV calling’ and highlighted by yellow color in our newly revised manuscript.
